# Primary Immunodeficiencies With Defects in Innate Immunity: Focus on Orofacial Manifestations

**DOI:** 10.3389/fimmu.2020.01065

**Published:** 2020-06-18

**Authors:** Sophie Jung, Vincent Gies, Anne-Sophie Korganow, Aurélien Guffroy

**Affiliations:** ^1^Université de Strasbourg, Faculté de Chirurgie Dentaire, Strasbourg, France; ^2^Hôpitaux Universitaires de Strasbourg, Centre de Référence Maladies Rares Orales et Dentaires (O-Rares), Pôle de Médecine et de Chirurgie Bucco-Dentaires, Strasbourg, France; ^3^Université de Strasbourg, INSERM UMR_S 1109 “Molecular ImmunoRheumatology”, Strasbourg, France; ^4^Université de Strasbourg, Faculté de Pharmacie, Illkirch-Graffenstaden, France; ^5^Hôpitaux Universitaires de Strasbourg, Service d'Immunologie Clinique et de Médecine Interne, Centre de Référence des Maladies Auto-immunes Systémiques Rares (RESO), Centre de Compétences des Déficits Immunitaires Héréditaires, Strasbourg, France; ^6^Université de Strasbourg, Faculté de Médecine, Strasbourg, France

**Keywords:** inborn errors of immunity, innate immunity, orofacial manifestations, oral management, primary immunodeficiencies

## Abstract

The field of primary immunodeficiencies (PIDs) is rapidly evolving. Indeed, the number of described diseases is constantly increasing thanks to the rapid identification of novel genetic defects by next-generation sequencing. PIDs are now rather referred to as “inborn errors of immunity” due to the association between a wide range of immune dysregulation-related clinical features and the “prototypic” increased infection susceptibility. The phenotypic spectrum of PIDs is therefore very large and includes several orofacial features. However, the latter are often overshadowed by severe systemic manifestations and remain underdiagnosed. Patients with impaired innate immunity are predisposed to a variety of oral manifestations including oral infections (e.g., candidiasis, herpes gingivostomatitis), aphthous ulcers, and severe periodontal diseases. Although less frequently, they can also show orofacial developmental abnormalities. Oral lesions can even represent the main clinical manifestation of some PIDs or be inaugural, being therefore one of the first features indicating the existence of an underlying immune defect. The aim of this review is to describe the orofacial features associated with the different PIDs of innate immunity based on the new 2019 classification from the International Union of Immunological Societies (IUIS) expert committee. This review highlights the important role played by the dentist, in close collaboration with the multidisciplinary medical team, in the management and the diagnostic of these conditions.

## Introduction

### Primary Immunodeficiencies (PIDs)/Inborn Errors of Immunity

Primary immunodeficiencies (PIDs) constitute a large and heterogeneous group of inherited conditions caused by germline mutations impairing or not protein expression, but resulting in either loss-of-function (LOF; hypomorphic [partial LOF] or amorphic [complete LOF]), or gain-of-function (GOF) of the encoded protein (1–4). To date, 406 distinct disorders have been described with 430 identified gene defects that affect the immune system development and/or function (3, 4). Although PIDs are still considered as rare diseases, epidemiologic studies have suggested that they may be underdiagnosed (5, 6) and their collective prevalence is now estimated to range between 1/1,000 and 1/5,000 (3). The main clinical feature of PIDs is an increased susceptibility to severe, atypical, persistent, and/or recurrent infections. According to the gene defect, the susceptibility can vary from a strong predisposition to a large variety of microorganisms, to a selective susceptibility to a single type of infection (1). However, the phenotypic spectrum of PIDs is extremely large and extends beyond infections. It includes a wide range of clinical manifestations associated with immune dysregulation such as allergy, auto-immunity/inflammation, lymphoproliferation, and malignancies (7–9). Considering the growing recognition of immune dysregulation, the denomination “primary immune deficiencies” appears too restricted and one should now use the terminology “inborn errors of immunity” (2). For simplicity, we will however use the abbreviation “PID” in this review. According to the 2019 classification of the International Union of Immunological Societies (IUIS) expert committee of Inborn Errors of Immunity, PIDs are divided into 10 categories based on shared pathogenesis and/or clinical phenotypes (Table S1) (3, 4).

### Oral Involvement in PIDs

PIDs can affect almost all organ systems and tissues including the orofacial region. Patients with compromised immunity, in particular when the innate immunity is affected, are therefore predisposed to a variety of oral manifestations including, among others, infections (e.g., candidiasis, herpes gingivostomatitis^*^, atypical dental infections), oral aphthous ulcers^*^, severe periodontal diseases^*^, and dental anomalies^*^. These lesions can in some cases be inaugural, preceding the other clinical manifestations and therefore being one of the first features of an underlying defect of immunity. In some PIDs of the innate immunity, they can even represent the main clinical manifestation, as in Papillon-Lefèvre syndrome (PLS). One of the most significant situation is the occurrence of aggressive periodontitis^*^ with premature tooth loss in children/adolescents as it almost always indicates the existence of an underlying systemic or immunologic disorder, in particular neutrophil defects. The medical team, and especially the dentists, should be aware of the main oral features associated with the different PID disease categories and should be able to identify atypical oral lesions that may signal a previously undiagnosed PID. Although often underestimated, the role played by dentists in the detection of warning clinical signs is crucial as it can contribute to a timely diagnosis and an early implementation of adequate management. Indeed, the latter is essential to avoid the persistence of infectious and inflammatory foci that may have an impact on the course of the PID itself (i.e., development of autoimmune manifestations, malignant transformation).

The aim of this review is therefore to describe the spectrum of orofacial features associated with the different PIDs of innate immunity and to give clues for the management of these manifestations. We focused on defects of phagocyte number and function (Category 5; Table 1) as oral manifestations are very prevalent. However, several other PIDs of intrinsic and innate immunity (Category 6; Table 1) are also characterized by specific oral manifestations such as oral candidiasis in chronic mucocutaneous candidiasis (CMC) disease for example. We will not discuss in this review the deficiencies of the complement cascade (Category 8; Table S1).

**Table 1:** Disease categories described in this review.

CATEGORY 5. Congenital defects of phagocyte number or function	1. Congenital neutropenias (CN)
	2. Defects of motility
	3. Defects of respiratory burst
	4. Other non-lymphoid defects
CATEGORY 6. Defects in intrinsic and innate immunity	1. Mendelian susceptibility to mycobacterial disease (MSMD)
	2. Epidermodysplasia verruciformis (EV)
	3. Predisposition to severe viral infection
	4. Herpes simplex encephalitis (HSE)
	5. Predisposition to invasive fungal diseases
	6. Predisposition to mucocutaneous candidiasis (CMC)
	7. TLR signaling pathway deficiency with bacterial susceptibility

*We followed the 2019 classification of the International Union of Immunological Societies (IUIS) (3)*.

## Methods: Search Strategy

We performed a literature review using PubMed/MEDLINE database (up to April 2020). Relevant articles were selected, based on combinations of MeSH or other search terms, without language or time restriction.

The terms “primary immune deficiency” OR “primary immunodeficiency” OR “inborn errors of immunity” AND “innate immunity,” or the terms referring to the different diseases (e.g., “severe congenital neutropenia,” “Papillon-Lefevre syndrome,” “chronic granulomatous disease,” “mendelian susceptibility to mycobacterial disease”), were used in combination with the following terms: “oral,” “oral mucosa,” “mouth,” “orofacial,” “teeth,” “oral ulcer,” “periodontal disease,” “periodontitis,” “candidiasis,” “gingivostomatitis.”

## Results

### Congenital Defects of Phagocyte Number or Function [Category 5 (3)]

Multiple genetic defects associated with a reduction in the absolute neutrophil count (ANC) or with an aberrant function of these cells are clinically characterized by recurrent infections due to extracellular pathogens. In addition, they are associated with very aggressive forms of periodontitis, which already present in early childhood or adolescence. As “gatekeepers of oral immunity,” fully functional neutrophils are essential in the maintenance of periodontal homeostasis (10). Indeed, they represent the majority of immune cells (>95% of total leucocytes) recruited to the gingival crevice^*^, also called gingival sulcus^*^, and form a “defense wall” against the subgingival dental biofilm^*^ (10–12) [for review see (11)].

### Severe Congenital Neutropenias

#### Genetic, Pathophysiology, and Clinical Manifestations

Severe congenital neutropenias (SCNs) represent a group of PIDs characterized by an impaired differentiation of neutrophils and an accumulation of atypical promyelocytes in the bone marrow due to maturation arrest of myelopoiesis (13). Consequently, patients present a severe chronic neutropenia (i.e., blood ANC below 0.5 × 10^9^ cells per liter or 500 cells per μl/mm^3^). The estimated prevalence of SNC ranges between 3 and 8.5 cases per million individuals (14). Already in the first months of life, SCN patients develop severe bacterial infections affecting the respiratory tract, the skin, and deep tissues and, to a lesser extent, fungal infections. One of the characteristic features is the absence of pus formation. In addition, SCN is considered as a pre-leukemic condition with a marked propensity for hematopoietic malignant transformation leading to acute myeloid leukemia (AML) and myelodysplastic syndromes (MDS) (13, 15).

Germline mutations have been identified in more than 20 genes (Table 2), but almost half of the patients carry heterozygous variants in *ELANE* gene (alternative name *ELA2*), which encodes neutrophil elastase (13).

**Table 2:** Characteristics of the different forms of severe congenital neutropenias (SCNs).

**Name**	**MIM code**	**Mutant gene**	**Inheritance**	**Function of the mutated protein**	**Effect of the mutation**	**Percentage of cases**	**Clinical features in addition to neutropenia and severe bacterial infections**
SCN1 or elastase deficiency (13)	202700	*ELANE or ELA2* (neutrophil elastase)	AD	Cytotoxic serine protease released upon neutrophil activation. Hydrolysis of various substrates (e.g., cell membrane proteins such as G-CSF receptor, extracellular matrix proteins, cell adhesion proteins). Role in neutrophils mobilization from the bone marrow	Intracellular accumulation and mislocalization of mutant neutrophil elastase ER stress leading to increased apoptosis (16).	~45%	Osteopenia
SCN2 (17)	613107	*GFI1* (Growth Factor Independent Protein 1)	AD	Transcriptional repressor oncoprotein regulating ELANE Role in the control of normal hematopoietic cell differentiation (18, 19)		<1% of identified germline mutations	Lymphopenia Increased number of circulating immature myeloid cells
SCN3 or “Kostmann syndrome” (20)First described in 1956 and called “infantile agranulocytosis” (21)	610738	*HAX1* (HCLS1 [Hematopoietic Cell-Specific Lyn Substrate 1] associated protein X-1)	AR	Mitochondrial protein with anti-apoptotic properties Binds to HCLS1, an essential adapter protein of G-CSF signaling pathway	Increased apoptosis Abrogated G-CSF signaling (20, 22)	~10%	Neurological phenotype in patients with mutations affecting both splice isoforms of *HAX1* (23)
SCNX or X-linked neutropenia/myelodysplasia (24–26)	300299	*WAS* (Wiskott-Aldrich syndrome)GOF germline mutations^†^	XL	WAS protein (WASP): regulator of actin filament reorganization	Constitutive activation of WASP Increased actin polymerization Aberrant cell division (25, 27)	~2%	Monocytopenia Myelodysplasia Lymphocytes anomalies (e.g., increased number of activated CD8+ T cells) (24, 28).
SCN7 or G-CSF receptor deficiency (29, 30)	617014	*CSF3R*(G-CSF receptor)	AR	G-CSF receptor	Absence of G-CSF receptors on the cell surface Impairment of G-CSF signal transduction	Very rare	SCN despite normal granulocyte maturation on bone marrow biopsies

*AD, autosomal dominant; AR, autosomal recessive; CID, combined immunodeficiency; ER, endoplasmic reticulum; G-CSF, granulocyte-colony stimulating factor; GOF, gain of function; SCN, severe congenital neutropenia; WAS, Wiskott-Aldrich syndrome; WASP, Wiskott-Aldrich syndrome protein; XL,X-linked*.

† *LOF germline mutations in WAS gene lead to Wiskott-Aldrich syndrome (WAS), an X-linked syndromic CID with congenital thrombocytopenia (31, 32)*.

Congenital neutropenia can also be found in association with additional immunologic and non-hematopoietic features in several syndromic disorders that are due to rare pathogenic variants in genes controlling ribosome maturation (e.g., *SBDS, DNAJC21*), lysosomal function (e.g., *LAMTOR2, VPS13B*), or glucose metabolism (e.g., *G6PC3, SLC37A4*), among others (13, 33) (Table 3).

**Table 3:** Characteristics of syndromic forms of congenital neutropenias.

**Name**	**MIM number**	**Mutant gene**	**Function of the mutated protein**	**Inheritance**	**Other hematological features**	**Extra-hematopoietic features (in addition to recurrent bacterial infections)**	**Other facial and oral abnormalities**
Barth syndrome or 3-methylglutaconic aciduria type II (34)	302060	*TAZ*	Acyltransferase tafazzin Involved in lipid metabolism and regulation of phospholipid membrane homeostasis	XL	None	Cardiomyopathy Skeletal myopathy Developmental delay (growth, delayed puberty) Increased urinary excretion of 3-methylglutaconic acid Attention deficit, mild learning disabilities	Facial features: deep set eyes, round face with full cheeks (“cherubic” appearance) during early childhood, prominent ears and narrow head and face after puberty (34) Oral mucosa: oral ulcers Others: oro-motor feeding problems (sensitive gag reflex, poor chewing skills)
Clericuzio syndrome (poikiloderma^*^ with neutropenia) (35, 36)	604173	*C16ORF57 (USB1)*	U6 snRNA phosphodiesterase Exoribonuclease involved in RNA processing from pre-RNA	AR	Possible evolution to MDS or AML (rare) Transient thrombocytopenia and variable anemia	Rare genodermatosis Inflammatory eczematous rash (age 6–12 months) followed by post-inflammatory poikiloderma^*^ (age >2 years) Other ectodermal findings: nail dystrophy (thickened nails), palmo-plantar hyperkeratosis, non-healing skin ulcers, calcinosis cutis Developmental delay (short stature, delayed puberty) Bronchiectasis	Characteristic facial features: prominent forehead, depressed nasal bridge, mid-facial hypoplasia, sparse eyebrows and eyelashes, dry and thin hair Teeth: delayed dental eruption (35, 36)
Cohen syndrome (37)	216550	*VPS13B (COH1)*	Vacuolar protein sorting-associated protein 13B Intracellular vesicle-mediated sorting and protein transport, Golgi complex integrity	AR	None	Early-onset hypotonia Truncal obesity Developmental delay (short stature) Psychomotor retardation, sociable behavior Severe myopia Joint hypermotility Small hands and feet	Characteristic facial features: microcephaly, hypotonic face, thick hair, low hairline, high-arched and wave-shaped eyelids, long and thick eyelashes, thick eyebrows, prominent, beak-shaped nose with a high nasal bridge, malar hypoplasia, smooth or short and upturned philtrum, maxillary prognathia/hyperplasia, high-arched palate, forward-inclined upper central incisors, “open-mouth” appearance (labial incompetence) Oral mucosa: oral ulcers (37–39) Teeth: agenesis^*^ (40)
Glucose-6-phosphatase 3 (G6PC3) deficiency (SCN4) (41, 42)	612541	*G6PC3*	Hydrolysis of glucose-6-phosphate to glucose and phosphate (43)	AR	Thrombocytopenia Evolution to MDS or AML	Prominent superficial venous pattern Congenital cardiac and urogenital malformations Endocrine abnormalities Skin hyper-elasticity	Minor facial dysmorphism: triangular shape of the face, depressed nasal bridge Cleft palate or high palate (41, 42)
Glycogen storage disease type 1b (44, 45)	232220	*SLC37A4* (*G6PT1*)	Glucose-6-phosphate exchanger Regulation of glucose-6-phosphate transport from the cytoplasm to the ER lumen Maintenance of glucose homeostasis and ATP-mediated calcium sequestration in the ER	AR	Impaired monocytes and platelets functions Evolution to MDS or AML	Metabolic disease: fasting hypoglycaemia, lactic acidosis, glycogen and fat accumulation in the liver leading to hepatomegaly, hyperlipidemia, IBD, pancreatitis Growth retardation (short stature, delayed puberty) Osteoporosis Thyroid autoimmunity	Facial features: full-cheeked round “doll-like” face Oral mucosa: hyperplastic/hypertrophic gingiva, giant cell granulomatous epulis (46), oral ulcers (aphtous gingivostomatitis) Teeth: delayed dental development and eruption (44–47), enamel hypomineralization (48) Others: feeding difficulties, orofacial myofunctional disorders, decreased taste perception (49)
JAGN1 deficiency (SCN6) (50)	616022	*JAGN1*	Jagunal homolog 1 Involved in early secretory pathway, cell adhesion and cytotoxicity	AR	Evolution to AML	Bone abnormalities (osteoporosis, thickening of skullbones)	Teeth: dental “malformations” in 2 patients, amelogenesis imperfecta in 1 patient (50)
3-methylglutaconic aciduria with cataracts, neurologic involvement, and neutropenia (MEGCANN) or 3-methylglutaconic aciduria type VII (51)	616271	*CLPB*	Caseinolytic peptidase B May function as a regulatory ATPase and be related to secretion/protein trafficking process	AR	Evolution to MDS or AML	Variable phenotype Infantile onset progressive encephalopathy/brain atrophy Delayed psychomotor development Cataract Epilepsy 3-methylglutaconic aciduria Cardiomyopathy	Facial features: microcephaly, low nasal bridge, hypertelorism, tented mouth (51) Feeding difficulties
P14/LAMTOR2 deficiency (52)	610798	*LAMTOR2*	Late endosomal/lysosomal adaptor, MAPK and mTOR activator 2 Involved in amino acid sensing and activation of mTORC1	AR	Hypogamma-globulinemia Decreased cytotoxicity of CD8+ T cells	Developmental delay (short stature) Skin hypopig mentation (partial albinism)	Coarse facial features (52)
Shwachman-Diamond syndrome (SDS) (53)	260400	*SBDS*	Central role in biogenesis and maturation of ribosomes	AR	Cytopenia (thrombocytopenia, anemia) Evolution to MDS or AML	Exocrine pancreatic insufficiency Skeletal abnormalities (chondrodysplasia) Developmental delay (short stature) Cardiomyopathy Hepatomegaly Possible neurodevelopmental delay	Teeth: delayed dental development Oral mucosa: oral ulcers (54)
		*DNAJC21*		AR			
		*EFL1*		AR			
		*SRP54* (55, 56) (SDS-like)		AD		Only in patients with pathogenic variants interfering with G4-G5 elements of SRP54 (56)	
SMARCD2 deficiency or specific granule deficiency 2 (SGD2) (57)	617475	*SMARCD2*	Involved in transcriptional activation and repression of select genes by chromatin remodeling Regulation of TFs during hematopoietic differentiation	AR	Hypercellularity of the bone marrow Abnormal megakaryocytes Progressive myelofibrosis with blasts Anemia, thrombocytopenia Early death withoutHSCT	Delayed separation of umbilical cord Developmental delay Dysplastic nails Mild distal skeletal anomalies Parasitic infections Chronic diarrhea	Teeth: malpositions, enamel hypoplasia Mild facial features: asymmetric face, low-set and posteriorly rotated ears with prominent concha, hypoplastic mandibula, saddle nose, midface hypoplasia, synophris^*^ (1 patient) (57)
VPS45 deficiency (SCN5) (58, 59)	615285	*VPS45*	Vacuolar protein sorting-associated protein 45 Role in vesicle-mediated protein trafficking (endosomal, lysosomal, through trans-Golgi network)	AR	Myelofibrosis with bone marrow failure leading to extramedullary hematopoiesis with nephromegaly and hepatosplenomegaly Lack of response to G-CSF, early death without HSCT	Possible neurological abnormalities Failure to thrive	Oral mucosa: candidiasis Facial features reported in one patient: round facies, prominent forehead, long almond-shaped palpebral fissures, bulbous nasal tip, short columella, thin upper lip (58).

AD, autosomal dominant; AML, acute myeloid leukemia; AR, autosomal recessive; ER, endoplasmic reticulum; G-CSF, granulocyte colony-stimulating factor; HSCT, hematopoietic stem cell transplantation; IBD, inflammatory bowel disease; MDS, myelodysplastic syndrome; SCN, severe congenital neutropenia; TFs, transcription factors; XL: X-linked;

* *see lexicon (Table S2)*.

#### General Management

Since the availability of recombinant granulocyte colony-stimulating factor (G-CSF), which is currently the treatment of choice for SCN, the quality of life of patients has improved significantly and the overall survival exceeds 80% (13, 60, 61). Administration of G-CSF results in an increase in the neutrophil count that is associated with a significant reduction in the number and severity of infectious episodes (13). However, in patients who do not respond to G-CSF treatment and/or develop secondary malignancies, hematopoietic stem cell transplantation (HSCT) remains the only available treatment option (62). Prolonged exposure to high dosage of G-CSF can result in the acquisition of somatic *CSF3R* mutations that generate truncated G-CSF receptors. This is responsible for an hypersensitivity to G-CSF with clonal proliferation favoring leukemic transformation (63). Indeed, patients who require higher doses of G-CSF have a cumulative incidence of leukemia of 40% after 15 years compared to 11% in patients that are more responsive to G-CSF (61). In G-CSF receptor deficiency (SCN7; Table 2), neutropenia is unresponsive to G-CSF, but granulocyte-macrophage colony-stimulating factor (GM-CSF) treatment may be effective (29, 30).

#### Oral Manifestations and Management

The main oral manifestations associated with SCN include recurrent oral ulcers and periodontal diseases (64). Considering the key protective role of neutrophils in the periodontal tissues, a reduction of neutrophil numbers at the gingival sulcus leads to a marked increase in host susceptibility to periodontal diseases (65). Indeed, patients with SCN often suffer from early onset aggressive periodontitis, affecting both the primary and the permanent dentitions, with intense inflammation and severe bone loss leading to premature tooth loss (66). Periodontal involvement has been already reported in the SCN family originally described by Kostmann (67). A diagnostic score has been develop in order to differentiate congenital from non-congenital neutropenia using data collected during the first consultation (68). Apart from the medical history and previous severe infections, the key factors for congenital neutropenia prediction include the oral features classically associated to defects in neutrophils numbers (i.e., oral ulcers and/or gingivitis^*^) (68). This highlights the important role of the dentists in the diagnosis of this disease entity. In addition, the levels of plasmatic pro-LL-37, the precursor of the antimicrobial peptide cathelicidin LL-37 that is crucial to control periodontal normal flora, represents an early marker of severe disease. Therefore, pro-LL-37 may be used as a fast and simple tool to facilitate differential diagnosis of chronic neutropenia and to discriminate SCN from autoimmune and idiopathic neutropenia (69).

Ye et al. studied the genotype-phenotype correlation between the mutated gene and periodontal involvement in 14 SCN patients (70). They observed that patients with pathogenic variants in *ELANE* present with more severe periodontal disease than patients with *HAX1* or unknown genetic defects. In addition, they found higher levels of the pro-inflammatory cytokine IL1β in the gingival crevicular fluid^*^ as well as a skewed microflora in the periodontal pockets^*^ of *ELANE* mutated patients (70). A correlation between the oral status and the treatment has also been studied. Several authors have observed that periodontal disease tends to persist in patients under G-CSF treatment, even after normalization of neutrophil counts (67, 70). In the family originally described by Kostmann, all surviving non-transplanted patients that have been treated by G-CSF presented periodontal disease (chronic gingival inflammation with or without bone loss) despite normal ANCs and prophylactic dental care (67). Pütsep et al. showed that G-CSF-treated SCN3 patients have proper numbers of circulating neutrophils but that these cells have an impaired production of the antimicrobial peptide cathelicidin LL-37 and its precursor (71). Professional periodontal maintenance, associated with strict oral hygiene, should therefore be continued even after normalization of ANCs in patients under G-CSF therapy (67). In some cases, the extraction of severely affected primary teeth could reduce the microbial load of periodontal pathogens and create a better environment for the eruption of permanent teeth. To date, HSCT remains the only available curative treatment of SCN and is associated with a stabilization of periodontal disease. Indeed, in the original Kostmann family, the patient who received HSCT had almost normal concentrations of LL-37 in neutrophils and saliva and no periodontal inflammation (67, 71).

### Cyclic Neutropenia

#### Genetic, Pathophysiology, and Clinical Manifestations

In addition to SCN1, heterozygous pathogenic variants in *ELANE* can cause cyclic neutropenia (CyN) (64, 72). CyN is a related disorder of granulopoiesis characterized by a regular oscillation in the number of circulating neutrophils (from normal levels to severe neutropenia) and other peripheral blood cells including monocytes, platelets, reticulocytes, and lymphocytes, usually with a 21-day periodicity. Fluctuations in blood cells numbers are due to an oscillatory production of cells by the bone marrow (64, 72). CyN is associated with a milder course of the disease and a lower risk of leukemic transformation. The main clinical manifestations that usually appear during early childhood include recurrent fever, painful oral mucosal ulcers as well as skin and oropharyngeal infections (64, 72). Between neutropenic intervals, affected individuals are usually healthy but life-threatening bacterial infections can occur during periods of severe neutropenia. Although neutrophil counts continue to cycle, clinical manifestations usually decrease in severity during adulthood (64, 72).

#### General Management

As for SCN, CyN management relies on G-CSF therapy.

#### Oral Manifestations and Management

The diagnosis of CyN is usually made based on a pattern of recurrent fever and oral ulcerations. Serial blood cell counts show regular oscillations with an average 3-week turnover frequency (64). Painful ulcerations that can affect any part of the oral mucosa during the neutropenic phases are often the initial manifestation of CyN, highlighting the crucial role of the dentist in the diagnosis of this condition. Severe periodontal diseases (i.e., gingivitis and periodontitis) also develop when ANCs are at their lowest point (73–77). In a recent systematic review, the authors reported oral ulcers in 18% of CyN patients but gingival inflammation was observed in almost all of the cases (65). Systemic symptoms such as fever usually decrease after puberty but adults with CyN continue to experience oral ulcers and periodontal disease (64, 70, 74). Although G-CSF therapy is associated with a reduction of oral ulcers (78), its combination with professional periodontal treatment and oral hygiene improvement is however required to control periodontal diseases.

### Defects of Motility

#### Leukocyte Adhesion Deficiency

##### Genetic, pathophysiology, and clinical manifestations.

The interaction of leukocytes with vascular endothelial cells, which is mediated by several families of adhesion molecules, is crucial for their migration to the tissues (79, 80). Leukocyte adhesion deficiency (LAD) is a group of autosomal recessive (AR) PIDs due to defects in leukocyte adhesion cascade with altered extravasation into tissues. To date, three different forms have been described (79). LAD type I [>320 reported cases (81)] affects the firm adhesion of leukocytes to the endothelium (79, 82, 83). LAD type II (<10 patients reported worldwide) is characterized by a defect in the rolling adhesion phase that involves transient interactions between P- and E-selectins (expressed by endothelial cells) with their fucosylated ligands (expressed by neutrophils) (79, 82, 84, 85). Finally, LAD type III (described in about 20 patients) is due to abnormal integrin activation that is crucial to induce the immobilization of neutrophils (82, 86) (Table 4).

**Table 4:** Features of the different forms of leukocyte adhesion deficiency (LAD).

**Name**	**MIM code**	**Mutant gene**	**Pathophysiology**	**Biological findings**	**Clinical features**	**Orofacial manifestations**	**General management**
LAD type I (LAD1) (81–83)	116920	*ITGB2*Encodes for common β2 subunit of integrin (CD18)	Defective binding between integrin α and β2 chains (CD18) **Defective firm/stable adhesion**	Blood hyperleucocytosisMarked granulocytosis during acute infectionAbsent/reduced CD18 expression at leukocytes' cell membraneDominant IL23/IL17 signature at inflamed sites (87)	Severe and recurrent bacterial infections → skin and mucosa ++ →*S. aureus*, Gram negative bacteria → absence of pus formation → severity directly correlated to the degree of CD18 deficiencyFungal infectionsDelayed separation of umbilical cordOmphalitisImpaired healing of traumatic and surgical wounds	Severe gingivitis and periodontitis with early tooth lossPersistent oral ulcers	2 forms: → severe (<2% CD18 expression): very poor prognosis without HSCT → moderate (2–30% CD18 expression): survival possible without HSCT but antibiotic therapy required Adjunctive IVIgs (88)
LAD type II (LAD2) or congenital disorder of glycosylation type IIc (CDG IIc) (79, 82, 84, 85)	266265	*SLC35C1*Encodes for specific guanosine diphosphate (GDP)-fucose transporterthat translocates GDP-fucose from the cytosol into the Golgi lumen	General defect in fucose metabolism Decreased expression of fucosylated glycoproteins including Sialyl-Lewis X antigen (CD15s) on leukocytes (ligand for endothelial selectins) **Defective rolling adhesion**	Blood hyperleukocytosisAbsent/reduced CD15s expression at leukocytes' cell membraneRare Bombay blood group (hh) phenotype due to absence of H antigen (that also incorporates fucose)	Recurrent bacterial infections → less severe than in LAD1 → absence of pus formationNormal separation of the umbilical cordSevere mental retardationGrowth retardation	Severe gingivitis and periodontitis with early tooth lossPersistent oral ulcersFacial dysmorphism: brachycephaly, low hairline, thick and sparse hair, coarse facial appearance, puffy eyelids, depressed nasal bridge, broad nasal tip, long upper lip, everted lower lip, high arched palate, protruding and large tongue, mandibular prognathism, short and webbed neckDelayed dental eruption (85, 89)	Fucose replacement therapy (90)Control of infection with antibiotics
LAD type III (LAD 3) (82, 86)	612840	*FERMT3*Encodes for kindlin-3 that is expressed in hematopoietic cells with a major role in the regulation of integrin activation	Severely impaired activation by chemokines of all major integrins expressed by leukocytes and plateletsFailure of leukocytes to arrest on endothelial integrin ligands **General defect of beta-integrins**	Blood hyperleukocytosisDefects in platelet aggregation	Similar phenotype than LAD1Osteoporosis-like bone featuresSevere bleeding tendency similar to Glanzmann thrombasthenia	Severe gingivitis and periodontitis with early tooth lossPersistent oral ulcers	Prophylactic antibioticsRepeated blood transfusionsHSCT: only curative therapyRecombinant factor VIIa successfully used to prevent and treat severe bleeding in 1 patient (91)

*GDP, guanosine diphosphate; HSCT, hematopoietic stem cell transplantation; IVIgs, intravenous immunoglobulins*.

##### General management

The general management of each form of LAD is described in Table 4.

##### Oral manifestations and management

As LAD type II and type III have been reported in less than 50 cases worldwide, the management of oral manifestations has been mainly described for LAD type I (LAD1) patients, in particular those with moderate form that is characterized by residual CD18 expression (2–30%). These patients usually survive childhood without HSCT but they present recurrent infections and immune-related lesions of the skin and mucosal surfaces (81). Oral involvement is observed in more than 50% of the patients with moderate LAD1 and includes periodontal diseases as well as recurrent and painful oral ulcers (81). Palatal ulcer with perforation has been reported in one LAD1 patient (92). Periodontitis is extremely aggressive and has a very early onset, affecting already primary teeth. It is characterized by an intense inflammation and a rapid loss of periodontal tissues including alveolar bone^*^ (66, 93). Periodontitis in LAD1 patients is mainly unresponsive to standard treatments (i.e., mechanical removal of tooth associated biofilm in combination with antibiotics), leading to premature tooth loss before young adulthood (66). Historically, LAD1-associated periodontitis has been attributed to defective neutrophil control of the periodontal infection. LAD subgingival microbiome is significantly different from the biofilm observed in healthy individuals and in individuals with localized aggressive periodontitis (94). Indeed, it is characterized by a reduced microbial diversity with a loss of health-associated microbial species and an over representation of periodontitis-associated species such as *Parvimonas micra, Porphyromonas endodontalis, Eubacterium brachy*, and *Treponema* species. *Pseudomonas aeruginosa* was also detected in LAD1 although it is a bacterial species that is not typically found in subgingival plaque (94). However, Moutsopoulos et al. have recently shown that LAD1 periodontitis does not represent a “raging infection” due to uncontrolled bacterial invasion of periodontal tissues but is rather caused by a dysregulated host inflammatory response, where the bacteria serve as initial triggers for local immunopathology (95–97). Indeed, the translocation of bacterial products such as lipopolysaccharide into the underlying tissues stimulates the local inflammatory response and the induction of IL23-mediated immunity. This leads to an excessive production of the proinflammatory and bone-resorptive cytokine IL17, implicating for the first time in humans the role of neutrophils in the regulation of IL17 responses (96). It has been shown that this cytokine is also overproduced in common forms of chronic periodontitis (98). This IL17 exaggerated response is mainly localized to the mucosal tissues. In the absence of tissue neutrophils, as in LAD1, the IL23 response fails to downregulate and continuously induces IL17 (66). Inhibition of the IL17 pathway in the murine model of LAD1 is associated with a reduction of both the inflammatory periodontal bone loss and the bacterial load. This suggests that dysregulated IL17-driven inflammation consecutive to impaired neutrophil recruitment fuels periodontal microbial overgrowth (95). Recently, the team of Moutsopoulos treated a patient with moderate form of LAD1, refractory periodontitis and non-healing cutaneous ulcers with anti-IL12/IL23 monoclonal antibody (ustekinumab) that inhibits IL23-dependent production of IL17 (87). The treatment allowed a significant reduction of oral inflammation and a complete resolution of the deep cutaneous wounds without significant adverse effect. Inhibition of IL23 and IL17 appears as a promising strategy in the management of moderate forms of LAD1, in particular for severe periodontal involvement (87), and an interventional protocol has been recently initiated (NCT03366142) (66). Indeed, despite strict oral hygiene regimen and regular periodontal treatment, most of the patients lose their teeth (99, 100). Their replacement relies on prosthetic rehabilitation with an age-specific approach. In growing patients, removable prostheses are favored and can be adapted depending on the teeth that are lost. To date, dental implants have been reported in only one patient with LAD (101).

#### Papillon-Lefèvre Syndrome (PLS)

##### Genetic, pathophysiology, and clinical manifestations

First described in a French family by the physicians Papillon and Lefèvre (102), Papillon-Lefevre syndrome (PLS; MIM 245000) is a rare AR condition characterized by the association of aggressive early-onset periodontitis and palmoplantar hyperkeratosis. The prevalence of PLS ranges between 1 to 4 cases per million individuals (103). PLS is caused by homozygous (2/3 of the cases) or compound heterozygous (1/3 of the cases) pathogenic variants in the gene *CTSC* that encodes a lysosomal cysteine protease called cathepsin C (CTSC) or dipeptidyl peptidase I (103, 104). CTSC is involved in posttranslational modification and activation of many serine proteases stored primarily in azurophilic granules from neutrophils (i.e., neutrophil elastase, cathepsin G, proteinase 3) (105). CTSC plays also a role in the activation of granzymes A and B in cytotoxic T lymphocytes (106). Whereas, mature neutrophils of PLS patients lack all serine proteases' activity, the latter is normal in immature neutrophils. PLS phenotype may therefore arise from functional defects affecting mature neutrophils within tissues. For example, they are incapable of producing neutrophil extracellular traps (NETs) in response to reactive oxygen species (ROS) (107). However, despite lack of active serine proteases in neutrophils and cytotoxic T lymphocytes from PLS patients, the associated immunodeficiency is remarkably mild as an only 15 to 20% of PLS patients are predisposed to recurrent bacterial infections (108). Most of these infections are mild skin pyodermas, but occasionally, severe and/or fatal pyogenic abscesses involving internal organs (i.e., liver abscesses) do occur (109).

In addition to activation of immune cells, the proteolytic activity of CTSC has also been proposed to play a role in epithelial differentiation and desquamation (110), likely explaining the skin phenotype that is dominated by palmoplantar hyperkeratosis. The latter can vary from mild psoriasiform scaly skin to overt hyperkeratosis. Keratosis can also affect other sites such as elbows and knees, and additional clinical findings may include intracranial calcifications, hyperkeratosis of the hair follicles, nail dystrophy, and hyperhidrosis (111).

##### Oral manifestations

**Figure 1:**
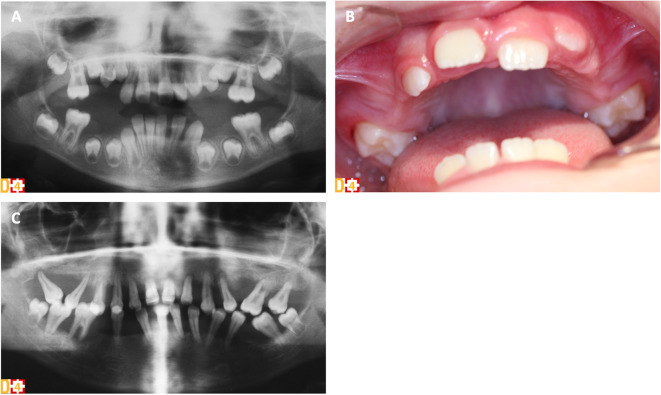
Clinical and radiological oral phenotype of two sisters with PLS syndrome (D[4]/Phenodent database (www.phenodent.org), Reference Center for Oral and Dental Rare Diseases, University Hospital, Strasbourg). **(A,B)** Premature tooth loss of the primary teeth in the 6-year-old patient. **(A)** Panoramic radiograph showing alveolar bone loss around the teeth, in particular at the mesial aspect of right permanent mandibular and maxillary first molars. **(B)** Intra-oral view showing the absence of all primary teeth. Absence of inflammation in the edentulous areas but presence of gingival inflammation around the erupting permanent maxillary first molars despite the perfect control of dental plaque. **(C)** Several periodontitis in the 16-year-old patient. Panoramic radiograph showing generalized severe vertical alveolar bone loss around all permanent teeth: typical radiological aspect of “floating” teeth.

Periodontitis in PLS patients is exceptionally severe with a very early-onset and a generalized pattern resulting in premature loss of both primary and permanent teeth (Figure 1). Although it has been associated with functional defects in neutrophils, the mechanisms by which CTSC deficiency leads to periodontitis have not been fully elucidated so far (66). Aggressive periodontitis in PLS patients could be attributed, at least in part, to a dysregulated inflammatory response rather than to an inefficient control of the periodontal bacteria. Indeed, CTSC deficiency results in failure to activate neutrophil-derived serine proteases, impairing proteolytic degradation of proinflammatory chemokines and cytokines, a mechanism important for periodontal tissue homeostasis (112). The subgingival microbiota in PLS patients is diverse with many periodontal pathogens commonly associated with chronic and aggressive periodontitis (e.g., *Aggregatibacter actinomycetemcomitans, Fusobacterium nucleatum*). Eruption of deciduous teeth occurs at expected ages with normal structure and form (113). Extremely intense gingival inflammation (e.g., erythema, edema, pain) develop shortly after eruption of primary teeth with a rapid periodontal destruction (e.g., deep periodontal pockets with pus exudate, extensive alveolar bone resorption, tooth mobility and migration) and premature tooth loss without signs of root resorption (Figures 1A,C) (111, 113, 114). Halitosis and lymphadenopathy are frequent. After the exfoliation or the extraction of the primary teeth, inflammation resolves rapidly. However, the cycle repeats itself with the eruption of permanent teeth (Figure 1B) (113). Radiological examination shows vertical alveolar bone loss around the teeth and in advanced stages, teeth can appear to be “floating” in the bone (Figure 1C) (113). Most PLS patients lose all of their primary and permanent teeth (66, 115) and only few patients without periodontal involvement have been reported (116, 117). Third molars remain however frequently unaffected (113). Gingival inflammation disappears in the totally edentulous patient. Although palmoplantar keratoderma and aggressive periodontitis are the cardinal clinical signs of PLS and usually manifest simultaneously between the age of 6 months and 4 years, no significant correlation has been found between the severity of these two conditions (118).

##### Skin and oral management

To date, therapeutic strategies remain limited and the management of both skin and oral manifestations is known to be difficult.

Regarding periodontal treatment, the main goal is to eradicate the reservoirs of periodontopathogens and to limit the destruction of periodontal tissues. The management of periodontitis includes careful plaque control with professional and individual oral hygiene regimens, the possible use of antiseptic mouth rinses (e.g., 0.2% chlorhexidine), conventional mechanical periodontal treatment to remove tooth-associated biofilm (scaling and root planing) along with courses of systemic antibiotics, followed by regular supportive periodontal therapy. The treatment of teeth with deep periodontal pockets may require flap surgical procedures (113, 119, 120). The commonly used systemic antibiotics are tetracyclines (that should be avoided in pediatric patients under 8 years of age due to the risk of teeth discoloration and enamel hypoplasia), erythromycin, amoxicillin plus metronidazole, or clavulanic acid (113, 119, 120). Extraction of primary teeth with poor prognosis, in combination with the eradication of the periodontal pathogens, creates a safe environment for the eruption of permanent teeth (113). However, despite regular and appropriate periodontal and antibiotic treatment regimens, the majority of the PLS patients lose all of their teeth. Nickles et al. analyzed long-term results (≥10 years follow-up) of periodontal treatment in eight patients with PLS and the teeth were maintained in only two of them (120). The replacement of lost teeth relies on prosthetic rehabilitation with an age-specific approach in a similar way than for LAD patients. Implants have been used by several authors in adult patients to improve stability and support of the prostheses (120–124). However, in PLS patients, implant placement is often complicated by the severe alveolar bone resorption due to early tooth loss. Although bone augmentation techniques and the use of short implants can possibly be considered (125), PLS patients are at high risk of peri-implantitis and implants loss. In Nickles et al. study, implants have been placed in four patients but three of them showed peri-implantitis only 4 years after their insertion (120). A very regular maintenance is therefore required to avoid early implant loss (120).

Treatment of dermatological manifestations relies on topical applications of emollients, keratolytic agents containing salicylic or lactic acid, and topical steroids to reduce skin inflammation. Several authors have suggested that oral retinoids such as acitretin, etretinate, and isotretinoin (analogs of vitamin A), which are effectively used in the treatment of various types of keratinizing disorders (by decreasing the keratin content of keratinocytes), may be beneficial in the management of cutaneous lesions of PLS, but also useful to prevent the loss of permanent teeth in children with PLS (126–131). Whereas, palmoplantar keratoderma usually improves rapidly in patients receiving oral retinoids, periodontal disease requires longer periods of treatment (128). The safety of oral retinoids in children remains however controversial due to their side-effects, in particular on skeletal development (132). Recently, an enzyme replacement therapy with recombinant CTSC has been developed and allowed to correct pathophysiologic markers in fibroblasts from PLS patients. It can therefore be a promising therapeutic approach for the future treatment of CTSC deficiency (133). A multidisciplinary team including dermatologists, pediatricians and dentists (periodontology, pediatric dentistry, oral surgery, prosthodontics) is important for the overall care of PLS patients.

#### *FPR1* Polymorphisms and Aggressive Periodontitis

Formyl peptide receptors (FPRs), which belong to a class of G-protein-coupled receptors, are highly expressed by neutrophils. FPR bind N-formylpeptides, produced by the degradation of bacterial cells, which are one of the major chemotactic stimuli guiding the migration of neutrophils to infection sites (134). This triggers an intracellular signaling cascade that coordinates cytoskeletal reorganization as well as the migration of neutrophils along the gradient of chemokines (134). Some studies highlight an association between *FPR1* (the gene encoding FPR) single nucleotide polymorphisms (SNPs) and aggressive forms of periodontitis that are rapidly progressing (135) [grade C periodontitis in the new classification of periodontal diseases (136, 137)]. rs5030879 (c.348 C>T) SNP was particularly studied. Indeed, it has been shown that African Americans with a homozygous 348T/T genotype exhibit a significantly lower neutrophil chemotactic response to formylpeptides than that observed in subjects with the 348T/C or 348C/C genotypes, with an increased risk to develop aggressive periodontitis (135, 138, 139).

#### WDR1 Deficiency

##### Genetic, pathophysiology, and clinical manifestations

Homozygous and heterozygous pathogenic variants in *WDR1* gene that encodes an actin-interacting protein alter the regulation of neutrophil cytoskeleton, causing neutrophil dysfunction with abnormal morphology (herniation of nuclear lobes), chemotaxis and survival (140, 141). A more recent study also identified defects in the T- and B-cell compartments (aberrant assembly of immunological synapses) (141). Patients present recurrent infections and varying clinical manifestations including mild neutropenia, skin ulceration, impaired wound healing, and moderate intellectual disability (140, 141).

##### Oral manifestations

Patients with WDR1 deficiency can suffer from severe aphthous stomatitis^*^ leading to oral stenosis, and from candidiasis. Facial dysmorphia has been observed in some patients (frontal bossing, hypertelorism, wide nasal nostrils) (141).

### Defects of Respiratory Burst

#### Chronic Granulomatous Disease (CGD)

##### Genetic, pathophysiology, and clinical manifestations

Chronic granulomatous disease (CGD) is a PID [~1 case in 200 000 to 250 000 live births (142, 143)] due to functional impairment of nicotinamide adenine dinucleotide phosphate (NADPH) oxidase in all phagocytes (neutrophils, monocytes, macrophages and dendritic cells [DCs]) (144). NADPH oxidase (NOX) is a multiprotein enzyme complex comprising both membrane-bound (cytochrome b558: gp91^*phox*^ and p22^*phox*^) and cytosolic (p40^*phox*^, p47^*phox*^, and p67^*phox*^) proteins that assemble upon phagocytes' activation. It catalyzes the transfer of electrons from NADPH to molecular oxygen to form superoxide ions that are used for the generation of ROS (e.g., hydrogen peroxide, hypochlorous acid). ROS production, called respiratory or oxidative burst, is a powerful antimicrobial mechanism essential for the destruction of phagocytosed bacteria and fungi (145). Pathogenic variants in the genes encoding any of the five structural subunits of NOX result in defective ROS production and in the development of CGD (144) (Table 5). More recently, it has been shown that homozygous germline mutations in *CYBC1* abolish the expression of EROS, a chaperone protein required for stable expression of membrane-bound components of NOX, and represent therefore a novel cause of CGD (148–150). Two thirds of CGD patients have an X-linked form with various germline mutations in *CYBB* (Table 5) (144, 151) and the majority of the patients are diagnosed during early childhood (151). Survival is associated with residual ROS production independently of the gene that is mutated (152). CGD patients suffer from a variety of severe and recurrent bacterial and fungal infections, in particular due to *Aspergillus* species, *Staphylococcus aureus, Burkholderia cepatia* species, *Serratia marcescens*, and *Nocardia* species (153, 154). The lungs (pneumonia), skin (abscesses, granulomas…), lymph nodes (lymphadenitis), and the liver (abscesses) are the most common affected sites (144, 151). In developing countries, BCG (Bacille-Calmette-Guérin) and *Mycobacterium tuberculosis* are also important pathogens (155). CGD is associated with a very high prevalence of invasive fungal infections that affect up to 40% of the patients and can be life-threatening (156). “Mulch pneumonitis,” due to an intense inflammatory response to fungal elements in aerosolized decayed organic matter, is almost pathognomonic of CGD (144, 151). In addition to severe infections, CGD patients also suffer from dysregulated inflammation, in particular in the gastrointestinal [inflammatory bowel disease (IBD)-like] and genitourinary tracts, and can develop granulomatous obstructive disorders (157). Inflammatory and autoimmune manifestations (e.g., arthritis, discoid lupus, systemic lupus erythematosus, vasculitis, immune thrombocytopenia) are observed in respectively 70 and 10% of the cases, with the highest frequency in X-linked CGD patients (142, 143, 157). As NOX is active in other cell types than phagocytes, the clinical picture of CGD may even be more complex (144).

**Table 5:** Germline mutations in NADPH oxidase complex leading to different CGD subtypes.

**Name**	**MIM number**	**Mutant gene**	**NADPH oxidase protein subunit**	**Inheritance**	**Localization of protein subunit at resting state**	**Percentage of cases**
CGD-X	306400	*CYBB* (cytochrome B-245 beta chain)	gp91*^*phox*^*/NOX2	XL	Membrane	65%
CGD4	233690	*CYBA* (cytochrome B-245 alpha chain)	p22*^*phox*^*	AR	Membrane	5%
CGD1	233700	*NCF1* (neutrophil cytosolic factor 1)	p47*^*phox*^*	AR	Cytosol	25%
CGD2	233710	*NCF2* (neutrophil cytosolic factor 2)	p67*^*phox*^*	AR	Cytosol	5%
CGD3	613960	*NCF4* (neutrophil cytosolic factor 4)	p40*^*phox*^*	AR	Cytosol	25 cases with an atypical form of CGD (hyperinflammation but no invasive infections) (146, 147)
No	No	*CYBC1* (cytochrome B-245 chaperone 1)	NA (the chaperone protein EROS (148) is necessary for stable expression of gp91*^*phox*^* and p22^phox)^	AR	Membrane	<5 reported cases (149, 150)

*AR, autosomal recessive; CGD, chronic granulomatous disease; NA, not applicable; XL, X-linked*.

##### General management

Diagnosis of CGD is made by functional evaluation of NADPH activity after phagocytes activation and by molecular confirmation. Conventional management predominantly relies on lifelong prophylactic antibiotics (trimethoprim-sulfamethoxazole) and antifungals (itraconazole), interferon (IFN)-γ therapy that can correct metabolic defects in phagocytes (158), along with the treatment of acute infections (144, 151). CGD-associated IBD is difficult to manage, in particular due to the combination with the inherent susceptibility to infections. The treatment of gastrointestinal (GI) tract inflammation frequently relies on corticosteroids although they remain controversial as they increase the growth retardation and the infectious risk (159). Allogeneic HSCT is currently the only curative treatment of CGD and may reverse both infectious and inflammatory manifestations (160).

##### Oral manifestations and gastrointestinal tract involvement

Several factors predispose CGD patients to oral manifestations. They include neutrophil dysfunction, but also the use of immunosuppressive therapies to manage inflammatory complications, as well as malnutrition due to GI complications. Indeed, GI tract inflammation is very frequent, with a reported incidence ranging from 30 to 60% (157, 159, 161, 162). Recent findings have demonstrated a crucial role of NOX complex in the regulation of gut immunity, regardless the susceptibility to infections (163). Although it remains a distinct entity, GI involvement in CGD mimics IBD with overlapping features of both ulcerative colitis and Crohn's disease (159). Every part of the GI tract can be affected from the oral cavity to the anus. In a series of 98 patients, the most frequent reported GI manifestation was non-infectious diarrhea, followed by oral ulcerations and anal fistulae (157). In an important cohort of 459 European CGD patients, oral ulcers have been observed in 11% of the cases (142) and in a long-term follow-up study on 39 patients, they have been reported in 26% of the cases, along with stomatitis (164). Oral ulcers are similar clinically to aphthae with frequent recurrences. Granulomatous inflammation of the oral mucosa with a nodular and cobblestoning aspect, which is typically observed in patients with Crohn's disease (orofacial granulomatosis), has however been rarely described in CGD patients (165). Most of CGD patients present very early-onset forms of IBD (159). Importantly, GI manifestations may precede the diagnosis of CGD as well as the development of infectious complications (166). CGD should be considered in all patients who present early-onset IBD. Recurrent oral ulceration can therefore represent one of the inaugural signs of the disease. To our knowledge, no cases of severe oral infection have been reported (167, 168). In the CGD cohort of Liese et al., only 5% of the patients had one episode of parotid glands infection during the 22-year follow-up (164). In addition, although some studies show that gingivitis is common in CGD patients, with a prevalence ranging from 11% (142) to 35% (169), severe periodontitis has been rarely observed (65, 165, 170). In a large survey on 368 CGD patients, severe gingival or periodontal inflammation has been found in only 2% of the cases (143). This is in contrast with other PIDs due to a defective function of neutrophils such as LAD1. One hypothesis of the reduced prevalence of periodontitis associated with this PID could be the absence of a respiratory burst in neutrophils, despite its importance in periodontal pathogens' destruction (65, 168). Indeed, enhanced ROS generation has been clearly involved in the pathophysiology of periodontal disease (168).

### Other Non-lymphoid Defects

#### GATA2 Deficiency

##### Genetic, pathophysiology, and clinical manifestations

LOF heterozygous pathogenic variants in *GATA2* (guanine-adenine-thymine-adenine 2), a zinc finger transcription factor regulating early hematopoietic differentiation as well as lymphatic and vascular development, cause *GATA2* haploinsufficiency (GATA2 deficiency: MIM 137295). Germline mutations arise spontaneously (*de novo*) but are then transmitted with autosomal dominant (AD) inheritance. The age of clinical presentation ranges from early childhood to late adulthood, with most of the cases occurring during adolescence and early adulthood. GATA2 deficiency is a protean disorder with a broad phenotype encompassing (I) multi-lineage cytopenia [DCs, monocytes, NK (natural killer) cells, B cells], (II) immunodeficiency with increased susceptibility to human papillomavirus (HPV), invasive non-tuberculous mycobacterial (NTM) and fungal infections, (III) high risk of developing hematologic malignancies (MDS/AML), (IV) pulmonary alveolar proteinosis (pulmonary disease), and (V) congenital lymphedema (vascular/lymphatic dysfunction) (171, 172). However, GATA2 deficiency has a variety of presentations and offers a challenge in any classification system. Indeed, a small proportion of patients present with only asymptomatic mild neutropenia and no other discernible hematological abnormalities except monocytopenia or macrocytosis, but with high risk of hematologic transformation (173).

##### General management

Allogeneic HSCT remains currently the best therapeutic option to prevent or treat hematologic malignancies and life-threatening opportunistic infections (174, 175).

##### Oral manifestations

Oral manifestations associated with GATA2 deficiency have not been reviewed extensively yet. Authors report infectious oral lesions mainly caused by HPV (i.e., warts, condylomas) but also by herpes simplex virus 1 (HSV-1). A close monitoring of these lesions is strongly required, in particular due to their high risk of HPV-related malignant transformation (i.e., squamous intra-epithelial lesions, Bowenoid papulosis^*^, invasive squamous cell carcinoma) (176, 177). Other oral features have been described and include recurrent ulcerations and blistering, gingival hyperplasia and inflammation, as well as glossitis (177, 178).

## Defects in Intrinsic and Innate Immunity [Category 6 (3)]

Defects in intrinsic and innate immunity are also characterized by several oral manifestations, in particular infections by various pathogens. However, in comparison with defects of phagocytes number and function, there are less data in the literature regarding the orofacial phenotypic spectrum as well as the oral management of these disorders.

### Mendelian Susceptibility to Mycobacterial Disease (MSMD)

#### Genetic, Pathophysiology, and Clinical Manifestations

Mendelian susceptibility to mycobacterial disease (MSMD) is primarily characterized by a selective predisposition to infections caused by atypical and weakly virulent mycobacteria such as *Mycobacterium bovis* BCG vaccines and environmental NTM. MSMD patients may also suffer from *bona fide* tuberculosis caused by *Mycobacterium tuberculosis*. Otherwise, they don't show obvious immunological abnormalities (179). However, MSMD designation does not recapitulate the whole phenotype of the patients as they also show increased susceptibility to certain intracellular bacteria, in particular *Listeria monocytogenes* and *Salmonella* species, and to mucocutaneous fungal infections due to *Candida* species. More rarely, other severe infections have been reported, but mostly in single patients. They include infections caused by intramacrophagic bacteria (e.g., klebsiellosis, nocardiosis), fungi (e.g., histoplasmosis, coccidioido-, and paracoccidioidomycosis), parasites (e.g., leishmaniasis), and even viruses (e.g., CMV, varicella-zoster virus VZV, human herpes virus-8 HHV8). Although mycobacterial diseases are by far the most common infections in these patients, it is now clearly recognized that the clinical phenotype of MSMD extends beyond them (179).

To date, MSMD has been diagnosed in more than 500 individuals worldwide with a prevalence of almost 1:50,000 (180). MSMD is caused by germline mutations affecting 16 different genes i.e., 14 autosomal genes [AD or AR inheritance: *IFNGR1* (181) and *IRF8* (182); AR inheritance: *IFNGR2* (183), *IL12B* (184)*, IL12RB1* (185)*, ISG15* (186)*, TYK2* (187)*, SPPL2A* (188), *IL12RB2* (189, 190)*, IL23R* (189, 190), *RORC* (191), *JAK1* (180), *IFNG* (192); AD inheritance *STAT1* (193)] and two X-linked gene [*NEMO* (194) and *CYBB* (195)]. Recently, AR complete IFNγ deficiency (pathogenic variants in *IFNG* encoding the IFNγ cytokine itself) has been described in MSMD patients (192). In addition, allelic heterogeneity at the different loci has led to the definition of 31 different genetic disorders (190). All disorders affect the IFNγ-mediated immunity, in connection with IL12/IL23/ISG15 immunity (190). MSMD is therefore a good example of PID with a relatively narrow infectious phenotype that originates from germline mutations involving molecules belonging to the same functionally connected immunological pathway. Indeed, IL12/IL23 dependent IFNγ mediated immunity is crucial for the control of intracellular pathogens, in particular mycobacteria (179, 190).

#### General Management

Considering the impairment of IFNγ immunity, recombinant IFNγ therapy should be considered as the “natural” treatment of MSMD. However, in patients with defects in IFNγ receptors (*IFNGR1* and *IFNGR2* pathogenic variants) that have complete lack of cellular responses to this cytokine, treatment with IFN-γ is not indicated (179). All patients require prolonged antibiotic treatments against mycobacteria and the other involved pathogens. BCG vaccination should be avoided. Abdominal surgery may be needed to remove the splenic and/or mesenteric lesions in some cases (179). HSCT remains the only curative treatment, especially for patients with severe forms of MSMD (196).

#### Oral Manifestations

##### Susceptibility to mucocutaneous candidiasis

The most common genetic etiology of MSMD is AR complete IL12 receptor β1 (IL12Rβ1) deficiency. The latter is due to germline mutations in *IL12RB1* gene that encodes one of the chains of IL12 and IL23 receptors (185). Mild forms of chronic mucocutaneous candidiasis (CMC) have been reported in about 25% of the patients (197–199). Whereas, IL12 is a key cytokine for IFNγ production, IL23 plays a role in the maintenance of IL17 producing T cells (Th17 cells) that are important effectors in host defense against fungi, in particular *Candida albicans* (200). Indeed, PID patients with impaired IL17 immunity [see “predisposition to mucocutaneous candidiasis (CMC)” section] are susceptible to *Candida* species and develop CMC (201). Ouederni et al. reported the clinical features of candidiasis in 35 patients with IL12Rβ1 deficiency and observed that recurrent oropharyngeal candidiasis was by far the most common presentation (in 34 patients). Although it was less severe than in patients with defects of IL17 axis, it tends to persist despite antifungal therapy (202). CMC is also observed in patients with pathogenic variants in *IL12B* gene, but not in other genetic etiologies of MSMD, as it is related to IL23-dependent impaired IL17 immunity. Indeed, *IL12B* encodes IL12p40, which is a common subunit of both IL12 and IL23 cytokines (179). However, patients with AR complete IL-12Rβ2 or IL23R deficiency that have been described more recently display mycobacteriosis without increased susceptibility to candidiasis (189). Bi-allelic germline mutations in *RORC*, which encodes RORγ and RORγT transcription factors, have been associated with impaired systemic IFNγ response to mycobacteria but also with defective IL-17 mucocutaneous immunity to *Candida*. RORγ- and RORγT-deficient individuals can therefore display both mycobacteriosis and mucocutaneous candidiasis (recurrent or persistent oral candidiasis in 70% of the cases) (191). MSMD must therefore been investigated in patients with CMC or persistent oropharyngeal candidiasis.

##### X-linked recessive MSMD type 1: *NEMO* pathogenic variants

NEMO (NF-κB essential modulator), also called IKBKG (inhibitor of NF-κB kinase regulatory subunit gamma) is an essential component of NF-κB (nuclear factor kappa B) signaling pathway. It is the regulatory subunit of the inhibitor of IκB kinase (IKK) complex, which activates NF-κB. Germline mutations in *NEMO* gene have long been known to cause different ectodermal dysplasia^*^ (EDA) syndromes i.e., *incontinentia pigmenti* (IP) and anhidrotic ectodermal dysplasia with immunodeficiency (EDA-ID) (Table 6). In its classical form, EDA is characterized by abnormalities of ectodermal structures including anodontia^*^ or oligodontia^*^ with cone shaped teeth, hypotrichosis, and hypohidrosis with heat intolerance (206, 207).

**Table 6:** Features of syndromes caused by germline mutations in NEMO other than XL-MSMD type 1.

**Name**	**MIM code**	**Mutation**	**Inheri-tance**	**Affected individuals**	**Effect of the mutation**	**Immunodeficiency**	**Other clinical features**
*Incontinentia pigmenti* (XD-IP) (203)	308300	Null mutations	XD	Females Lethal *in utero* in males	Abolition of NEMO-dependent NF-κB activation	No	Cutaneous lesions: neonatal bullous rash along Blaschko's lines followed by verrucous plaques and hyperpigmented swirling patterns Typical developmental features of EDA (abnormalities of ectodermal structures, e.g., hypodontia^*^, hypohidrosis) Ophthalmologic and CNS abnormalities
Anhidrotic EDA with immunodeficiency (XR-EDA-ID)	300291 300301 300584	Hypomorphic mutations	XR	Males	Impairment of NF-κB signaling	Increased susceptibility to a wide range of pathogens (pyogenic bacteria, mycobacteria, viruses) Invasive pneumococcal disease++	Developmental features of EDA in most cases (MIM 300291) (204) Osteopetrosis and lymphedema in addition to EDA features in some patients (OMIM 300301) ID without EDA typical features in some cases (MIM 300584) (205)
XR-MSMD type 1 (2)	300636	Hypomorphic mutations	XR	Males	Selective impairment of CD40-NEMO-NF-κB signaling pathway → impaired production of IL12 by monocytes → impaired IFNγ-mediated immunity	Mycobacterial diseases: *Mycobacterium avium* complex++	No typical features of EDA in most cases Only some patients with hypodontia or conic shaped teeth

CNS, central nervous system; EDA, ectodermal dysplasia; ID, immunodeficiency; MSMD, Mendelian susceptibility to mycobacterial disease; XD, X-linked dominant; XR, X-linked recessive; IP, incontinentia pigmenti;

* *see lexicon (Table S2)*.

In the 2017 IUIS classification (2), hypomorphic mutations in *NEMO* gene were classified in MSMD disease category as they were previously shown to cause also X-linked recessive (XR; MIM 300636) MSMD (type 1) (208). These pathogenic variants interfere selectively with the CD40-NEMO-NF-κB signaling pathway, leading to an impaired T-cell dependent production of IL12 by monocytes and monocyte-derived DCs in response to CD40 (194). As a result of impaired IFNγ-mediated immunity, infections are mostly limited to mycobacterial diseases, in particular due to *Mycobacterium avium* complex. Unlike other patients with germline *NEMO* mutations, most of XR-MSMD type 1 patients lack the developmental features typical of EDA. Only some cases have been reported to have hypodontia^*^ or conic shaped teeth, but to date, none of them has been described with oligo- or anodontia (180, 194, 208). In the most recent 2019 IUIS classification, *NEMO* mutations have however been removed from MSMD disease category and classified only as combined immunodeficiencies (CIDs) with syndromic features [disease category 2 (3)].

##### X-linked recessive MSMD type 2: *CYBB* pathogenic variants

As discussed before (see section on CGD), germline mutations in *CYBB* are responsible for the most common form of CGD (MIM 306400) (143, 144). More recently, specific pathogenic variants in *CYBB* have been associated with X-linked MSMD (type 2) in male subjects suffering from recurrent mycobacterial diseases. The MSMD-causing mutations in *CYBB* selectively affect the respiratory burst in macrophages that is crucial for protective immunity to mycobacteria. Unlike CGD patients, NADPH activity is normal in neutrophils and monocytes (195, 209). To our knowledge, no specific oral manifestations have been reported in these patients.

### Epidermodysplasia Verruciformis

#### EVER1-EVER2-CIB1 Deficiencies

##### Genetic, pathophysiology, and clinical manifestations

Epidermodysplasia verruciformis (EV) is a rare genodermatosis characterized by a selective susceptibility to keratinocyte-tropic HPV infections (subgroup B1) and that typically presents in early childhood (210, 211). Disseminated, flat, wart-like hypo-, or hyper-pigmented papules develop on the trunk, the neck, the face, the head as well as the extremities and are mainly benign. Lesions with greater malignant potential present as verrucous or seborrheic keratosis-like lesions and occur more frequently on sun exposed surfaces. Indeed, patients with EV have a higher risk to develop actinic keratosis and non-melanoma skin cancers, in particular cutaneous squamous cell carcinomas (210, 211). Homozygous LOF pathogenic variants in *EVER1* (MIM 226400) and *EVER2* (MIM 618231) genes, also named *TMC6* and *TMC8*, respectively, have been reported in ~75% of patients with EV (210, 211). More recently, biallelic germline mutations in *CIB1* gene (calcium- and integrin-binding protein-1) have also been described (212). CIB protein forms a complex with EVER1 and 2 and it has been suggested that the disruption of the CIB1–EVER1–EVER2-dependent keratinocyte-intrinsic immunity may underly the selective susceptibility to beta-HPVs of EV patients (212).

##### General management

The development of EV lesions cannot be prevented, but regular monitoring and appropriate treatment of skin lesions (e.g., surgical excision, cryotherapy) that might transform into skin cancers are recommended (210).

##### Oral manifestations

To our knowledge, no HPV-related lesions or cancers have been described in the oral cavity, but they can develop on the facial skin.

#### WHIM Syndrome

##### Genetic, pathophysiology, and clinical manifestations

WHIM syndrome (MIM 193670) is a rare AD condition whose incidence is estimated to be about 1 in 4.3 millions live births (213, 214). The term “WHIM” is an acronym of the main clinical manifestations including warts, hypogammaglobulinemia, infections, and myelokathexis (i.e., bone marrow retention). WHIM is caused by dominant heterozygous GOF pathogenic variants in the gene encoding the CX chemokine receptor 4 (CXCR4). Since CXCR4 is involved in the retention of neutrophils in the bone marrow, GOF germline mutations will exaggerate this process, thereby retarding neutrophil egress, leading to neutropenia (214). WHIM is mainly characterized by susceptibility to extensive HPV infection, which causes multiple cutaneous, plantar, anogenital, and oral warts. Warts can also occur in atypical locations such as the limbs, chest and face, and are unusually resistant to destructive treatments such as cryotherapy or surgery. HPV-driven squamous cell carcinomas constitute a significant cause of morbidity. A regular monitoring of HPV lesions, especially in the mouth and anogenital regions, is therefore strongly required and must include frequent biopsies (211, 214). Patients present severe neutropenia, but also often lymphopenia and monocytopenia, as well as moderate hypogammaglobulinemia, and suffer from frequent oto-sinopulmonary infections. Recurrent lung infections can lead to bronchiectasis and be associated with colonization by *Pseudomonas aeruginosa* and *Stenotrophomonas maltophilia*. Nasal and skin infections due to *Staphylococcus aureus* or *Streptococcus sp*. are also reported and can lead to skin abscesses, cellulitis or even septicemia (arthritis, osteomyelitis) (214).

##### General management

The management of WHIM syndrome includes HPV vaccination (although WHIM patients respond less robustly than healthy individuals), prophylactic antibiotics, intravenous or subcutaneous immunoglobulins (IV Igs/SC Igs) substitution to prevent oto-sinopulmonary infections, as well as G-CSF injections to enhance the release of neutrophils from the bone marrow (214). More recently, some patients have been treated with low-dose plerixafor, a CXCR4 antagonist, with encouraging results (NCT02231879) (215).

##### Orofacial manifestations

In addition to oral warts and HPV-related oral squamous cell carcinomas described above (216), patients with WHIM syndrome can present severe pyogenic bacterial infections (e.g., cellulitis, recurrent acute and chronic sinusitis), or viral (e.g., HSV-1, VZV) infections (217, 218). The development of early-onset periodontitis has also been reported in several WHIM patients with rapid progression leading to early tooth-loss (219). Aphthous ulcers can be observed but they are less common than in individuals with SCN (214, 220). Rarely, odontogenic infections may disseminate and cause brain abscess or endocarditis, in particular in cases of associated congenital cardiopathies (e.g., tetralogy of Fallot) that are common in WHIM patients (214). The impact of G-CSF or plerixafor treatment on periodontitis evolution has however not been assessed yet (66).

### Predisposition to Severe Viral Infection

#### Germline Mutations Affecting Interferon (IFN) Signaling Pathway

##### Genetic, pathophysiology, and clinical manifestations

IFN signaling is crucial for the defense against viral infections. Several gene defects affecting this pathway have been described (Table 7) and predispose to severe, even life-threatening, viral infections (e.g., encephalitis, pneumonitis), in particular due to herpes (e.g., HSV-1, VZV [varicella-zoster virus], CMV) and influenza viruses. As IFN signaling is also a central aspect of the response to other intracellular pathogens in macrophages and neutrophils, patients may in addition be susceptible to mycobacterial infections (239).

**Table 7:** Germline mutations affecting interferon signaling pathway and associated with a predisposition to severe viral infections.

**Name**	**MIM code**	**Mutant gene**	**Inheritance**	**Function of the mutated protein**	**Clinical features**
Complete STAT1 deficiency (221–224)	613686	*STAT1* (signal transducer and activator of transcription 1) LOF pathogenic variants^†^	AR	Key transcription factor mediating both type I (IFN-α and IFN-β) and type II (IFN-γ) IFN signaling Involved in immune response to viruses	Early disseminated mycobacterial and viral infections that are rapidly fatal
STAT2 deficiency (225, 226)	616636	*STAT2*	AR	Forms a complex with STAT1 and IRF-9 in response to IFNs Acts as a transactivator	Described in <10 patients Some patients remained asymptomatic whereas other presented severe viral infections (e.g., disseminated vaccine-strain measles following routine immunization)
IFNAR1 and IFNAR 2 deficiencies (227, 228)	616669(*IFNAR2*)	*IFNAR1* and *IFNAR2* (IFN-α/β receptors 1 and 2)	AR	Receptors that bind type I IFNs Downstream activation of JAK/STAT signaling	Severe complication following vaccination (IFNAR1 deficiency: yellow fever and measles; IFNAR2 deficiency: measles/mumps/rubella). Otherwise healthy individuals
IRF7 and IRF9 deficiencies (227, 229, 230)	616345 (*IRF7*) 618648(*IRF9*)	*IRF7* and *IRF9* (IFN regulatory factors 7 and 9)	AR	Belong to JAK/STAT signaling pathway Regulate the transcription of IFN	Life-threatening influenza infectionsOtherwise healthyindividuals
MDA5 deficiency (231–233)	/	*IFIH1* (IFN induced helicase C domain containing protein1)	AR LOF	Encodes MDA5, a cytoplasmic viral RNA receptor activating type I IFN signaling	Life-threatening susceptibility to common respiratory RNA viruses (e.g.,rhinoviruses)
RNA Polymerase III deficiency (234)	/	*POLR3A* *POLR3C* *POLR3F* (RNA polymerase III subunits A, C and F)	AD	Cytosolic DNA sensor activating type I IFN signaling	Severe primary VZV infection of the CNS and lungs Otherwise healthy individuals

*AD, autosomal dominant; AR, autosomal recessive; GOF, gain of function; IFN, interferon; IFNAR, interferon-α/β receptor; IRF, IFN regulatory factor; JAK, Janus kinase; LOF, loss of function; STAT, signal transducer and activator of transcription 1; VZV, varicella-zoster virus*.

† *Heterozygous GOF germline mutations in STAT1 gene are associated with chronic mucocutaneous candidiasis disease (235–238)*.

##### Oral manifestations

Viral infections, in particular due to herpes viruses, can manifest in the oral cavity. However, considering the severity of the infections affecting the patients with pathogenic variants in IFN signaling pathway, their oral localization is rarely mentioned. HSV-1 gingivostomatitis has been described in STAT2 deficient patients (225, 226) but to our knowledge, specific oral features have been reported neither in IFNAR1/2, IRF7/9, MDA5, nor in RNA polymerase III-deficient patients.

#### CD16 Deficiency

##### Genetic, pathophysiology, and clinical manifestations

FcγRIIIA (CD16) is a low-affinity receptor for IgG Fc that is expressed by NK cells. Homozygous pathogenic variants in the *FCGR3A* gene lead to CD16 deficiency (MIM 615707) characterized by functional deficiency of NK cells with defective cytotoxic activity but retained antibody-dependent cellular cytotoxicity. Patients typically present early in childhood with severe herpes viral infections, in particular due to Epstein Barr Virus (EBV), VZV, and HPV (240–242).

##### Oral manifestations

Patients are prone to HSV-1 gingivostomatitis and require regular monitoring of the oral mucosa (240–242).

### Herpes Simplex Encephalitis (HSE)

#### Genetic, Pathophysiology, and Clinical Manifestations

Pathogenic variants in genes that encode proteins belonging to the TLR3 signaling pathway (TRIF-dependent) result in early susceptibility to HSV-1 encephalitis (HSE) (243, 244). The spectrum of infections affecting the patients with TLR3 pathway defects is remarkably restricted to only one specific pathogen (HSV-1) and one specific type of infection (encephalitis).

#### Oral Manifestations

Surprisingly, children with HSE do not show an increased susceptibility to HSV-1-related diseases affecting other sites than the central nervous system (CNS) including herpes gingivostomatitis, which is the most common clinical symptom of HSV-1 infection in the general population (244, 245).

### Predisposition to Invasive Fungal Diseases

#### CARD9 Deficiency

##### Genetic, pathophysiology, and clinical manifestations

CARD9 (caspase recruitment domain-containing protein 9) is an adaptor molecule expressed principally in myeloid cells downstream from C-type lectin receptors activation (e.g., Dectin-1) by fungal ligands. Activated CARD9 couples with BCL10 and MALT1, resulting in NF-κB and mitogen-activated protein kinases (MAPK) activation. This signaling pathway promotes the production of key cytokines (e.g., IL1β, IL6, IL23) for antifungal immune responses (246, 247). CARD9 deficiency (MIM 212050) is characterized by the spontaneous development of invasive fungal infections due to fungi belonging the phylum *Ascomycota*. They include CMC (see “predisposition to CMC” section), invasive *Candida* infections (in particular of the CNS but also of the eyes, the colon and the bones), extensive and/or deep dermatophytosis, subcutaneous and invasive phaeohyphomycosis, as well as extrapulmonary invasive aspergillosis (248–250). CARD9 deficiency is the consequence of homozygous or compound heterozygous LOF germline mutations in *CARD9* that induce impaired cytokine production in response to fungal ligands, altered neutrophil killing and/or diapedesis, and defects of Th17 immunity. To date, more than 60 cases have been described with a very heterogeneous age of disease-onset ranging from childhood to adulthood (248–250).

##### General management

The treatment of patients with CARD9 deficiency is empirical, mainly based on antifungal therapies (e.g., azole agents, echinocandins) and on the surgical removal of fungal masses. In addition, CARD9-deficient patients should be given secondary prophylaxis with oral azole agents after the first episode of invasive fungal disease. In cases of persistent or relapsing *Candida albicans* infections of the CNS, adjuvant GM-CSF/G-CSF therapy can be considered. The potential value of HSCT still remains unclear due to the lack of available data (249).

##### Oral manifestations and management

Oral candidiasis (that is part of CMC due to *Candida albicans*) is very frequently associated to CARD9 deficiency and may reveal the disease (249, 251). It affects almost 30% of the patients as reported in a recent review (249). The use of oral fluconazole prophylaxis should also prevent the occurrence of CMC. In addition, frequent rigorous screening of the oral mucosa is required in order to diagnose CMC occurrence and/or relapse (249).

### Predisposition to Mucocutaneous Candidiasis (CMC)

#### Genetic, Pathophysiology, and Clinical Manifestations

Chronic mucocutaneous candidiasis (CMC) is characterized by recurrent or persistent symptomatic mucocutaneous infections caused by fungi from the *Candida* genus, in particular the commensal *Candida albicans*. CMC affects the nails, the skin, but also the genital and the oral mucosae (oral candidiasis) (252). The term CMC disease (CMCD) is used to refer to patients presenting with CMC as the major clinical phenotype, with neither invasive fungal infections, nor other overt infectious or autoimmune manifestations (252, 253). Four of the five causative genes of CMCD are directly involved in IL17 signaling and encode the cytokine IL17F (*IL17F*), IL17 receptors (*IL17RA* and *IL17RC*) (201, 254), and ACT1 (*TRAF3IP2*), a membrane-proximal adaptor of IL17 receptor (255, 256) (Table 8). The discovery of these genetic defects highlighted the essential role of IL17 cytokines for mucocutaneous protection against *Candida albicans*. Heterozygous GOF germline mutations in *STAT1* account for more than a half of CMCD cases (235–238). AD *STAT1* GOF leads to defective Th1 and Th17 responses, with reduced production of IFN-γ, IL17, and IL22 (236). However, more recent studies revealed that *STAT1* GOF pathogenic variants are associated with an unexpectedly wide range of clinical manifestations in addition to CMC. They include other infectious manifestations such as bacterial infections of the skin and the respiratory tract (*Staphylococcus aureus*), herpes virus infections (e.g., HSV-1, VZV), invasive fungal infections, mycobacterial disease, but also various autoimmune manifestations (>30% of the patients), cerebral aneurysms and malignancies, the latter two conferring a poor prognosis (237, 238). Very recently, AD germline mutations in the *MAPK8* gene encoding the kinase JNK1 have been reported in a family with a combination of CMC and a previously undescribed form of connective tissue disorder resembling Ehlers-Danlos syndrome (257). JNK1 haploinsufficiency impairs both IL-17–dependent mucocutaneous immunity to *Candida* and TGFβ-dependent homeostasis of connective tissues (257).

**Table 8:** Germline mutations leading to chronic mucocutaneous candidiasis disease (CMCD).

**Disease name**	**Mutant gene**	**Encoded protein**	**MIM code**	**Inheritance**
IL17F deficiency (201)	*IL17F*	Cytokine IL17F (member of IL17 family)	613956	AD
IL17RA deficiency (201)	*IL17RA*	IL17 receptor A	613953	AR
IL17RC deficiency (254)	*IL17RC*	IL17 receptor C	616445	AR
ACT1 deficiency (255)	*TRAF3IP2*	Adaptor protein ACT1	615527	AR
STAT1 GOF (235, 236)	*STAT1*	STAT1 transcription factor	614162	AD
JNK1 deficiency (257)	*MAPK8*	JNK1 kinase	No	AD

*AD, autosomal dominant; AR, autosomal recessive*.

#### General Management

Most patients with CMCD are treated with a combination of topical and systemic antifungal agents, in particular azoles (fluconazole in first line, followed by itraconazole, posaconazole, and/or voriconazole) (253). They require long-lasting antifungal treatments and/or prophylaxis to manage persistent and prevent recurrences (253). Clinical resistance to at least one antifungal agent has however been observed in almost 40% of *STAT1* GOF patients with long-term treatments (237). Topical therapy with polyenes (i.e., nystatin) has been proven to be a good alternative to triazoles (238). Oral ruxolitinib, a JAK1/2 kinase inhibitor that limits STAT1 mediated intracellular signaling, seems promising and allowed an improvement of CMC associated to *STAT1* GOF. However, long-term administration seems necessary, as the effect is not sustained after treatment discontinuation (258, 259). HSCT might be considered in STAT1 GOF patients with progressive life-threatening disease unresponsive to conventional treatments (260). Finally, recombinant IL17 may also represent a promising therapeutic option.

#### Oral Manifestations

Considering the high susceptibility to *Candida albicans* mucosal infections associated with IL17 axis disruption, almost 100% of CMCD patients present recurrent/persistent and/or severe candidiasis of the oral mucosa (thrush, glossitis, and/or cheilitis) (253). Germline mutations affecting IL17 signaling pathway should therefore been investigated in patients with recurrent oral candidiasis. One third of patients with *STAT1* GOF pathogenic variants also suffer from mucocutaneous viral infections including HSV-1 gingivostomatitis (237, 261). The treatment relies on appropriate systemic/topic antifungal and systemic antiviral agents.

In addition, CMCD patients, in particular those bearing AD *STAT1* GOF pathogenic variants, present an increased risk of both oral and esophageal squamous cell carcinoma, in part due to chronic inflammation associated with persistent CMC (237, 262, 263). Regular examination of the oral mucosa is therefore required.

Oral anomalies have also been described and include progressive macroglossia, macrocheilitis, as well as dental abnormalities (peg-shaped incisors) in ACT1 deficiency (255, 264). Delayed exfoliation of primary molars and enamel erosions have been reported in only one patient with *STAT1* GOF mutation (265). Although STAT1 has been involved in enamel formation in the rat (266), the link between *STAT1* germline mutations and the observed tooth anomalies (that are frequently observed in the general population) needs to be further studied. Delayed exfoliation of primary teeth and dental crowding that have been reported in the family with JNK1 haploinsufficiency may be related to impaired TGFβ-dependent homeostasis of connective tissues (257).

### TLR Signaling Pathway Deficiency With Bacterial Susceptibility

#### IRAK-4 and MyD88 Deficiencies

##### Genetic, pathophysiology, and clinical manifestations

AR IRAK-4 (IL-1 receptor-associated kinase-4) and MyD88 (myeloid differentiation factor 88) deficiencies selectively impair the signaling via the TLR and IL1 receptor pathway (267, 268). To date, more than 80 patients have been diagnosed worldwide [reviewed in (268)]. IRAK-4 (MIM 607676) and MyD88 (MIM 612260) deficiencies, which are phenocopies in term of clinical and immunological abnormalities, are characterized by a selective predisposition to pyogenic bacterial infections (267–270). Patients are highly susceptible to invasive bacterial infections caused by *Streptococcus pneumoniae* and, to a lesser extent, *Staphylococcus aureus*, as well as to non-invasive bacterial infections mainly restricted to the skin (*Staphylococcus aureus*) and the upper respiratory tract (*Pseudomonas aeruginosa*). Despite a large impact of these genetic defects on immune responses, affected individuals however present a normal resistance to common viruses, fungi, parasites and to many bacteria (270).

##### General management

In addition to vaccinations, in particular against *Streptococcus pneumoniae*, prophylactic antibiotic treatment (cotrimoxazole plus penicillin V) should be taken throughout the life. IV or SC Igs administrations during infancy seem to decrease the incidence of invasive bacterial infections (268). Empirical parenteral antibiotic treatment against *Streptococcus pneumoniae, Staphylococcus aureus*, and *Pseudomonas aeruginosa* must be initiated as soon as an infection is suspected, considering the high mortality risk due to invasive bacterial infections. Secondary adaptation of the treatment should then be done once the causal bacteria has been evidenced (270).

##### Oral manifestations

Bacterial infections involving the orofacial region such as maxillary sinusitis, necrotizing palate infection, cellulitis, and periodontal diseases have been reported in some patients with IRAK-4 and MyD88 deficiencies (268). Four patients also presented oral candidiasis (268). The treatment relies on appropriate systemic antibiotics and systemic/topic antifungal agents, respectively. Into et al. have shown in a murine model that MyD88 deficiency has an impact on the expression of several antimicrobial factors, which could also influence the susceptibility to oral infections (271).

## Discussion

Patients with inborn errors of innate immunity are prone to several kind of fungal, viral and/or bacterial infections that frequently involve the oral cavity. Lesions affecting the oral mucosa and the periodontium^*^ such as aphthous oral ulcers and early-onset aggressive periodontitis [grade C periodontitis in the new classification of periodontal diseases (136, 137)] respectively are also commonly observed, in particular in patients with defects of neutrophil number or function. Moreover, some PIDs of innate immunity, especially syndromic SCN (Table 3), are associated with developmental abnormalities including facial, oral, and even dental anomalies. Despite the large constellation of orofacial features, oral examination is sometimes overlooked from the global physical examination of the patients with a suspicion of PID, possibly because of a lack of habit or because the PID multidisciplinary team rarely involves dentists. In addition, dentists are not always familiar with these disorders. As a consequence, oral manifestations associated with inborn errors of immunity are frequently on the sideline and overshadowed by the other medical problems. However, oral features are of prime importance as they may reveal an underlying defect of immunity or further complicate the medical management of PID patients (see warning oral signs, Box 1). Indeed, they can promote a pro-inflammatory situation, lead to infectious complications, or even oncogenic transformation due to defective pathogen control (e.g., HPV). Both physicians and dentists should therefore be aware of the oral warning signs and the basic principles of oral management.

Box 1 **Warning oral signs.**

- Severe gingivitis in children that persists despite dental plaque/biofilm removal/control.
- Early-onset periodontitis with premature loss of primary teeth in children or loss of permanent teeth in adolescent/young adults.
- Aggressive forms of periodontitis that do not respond to conventional periodontal treatments.
- Recurrent and/or persistent oral ulcers.
- Severe and/or recurrent and/or persistent forms of herpetic gingivostomatitis or oral candidiasis that do not respond to treatment.
- Severe and/or atypical (involving uncommon pathogens) dental infections.
- In particular in children/adolescents.

Preventive measures including strict oral hygiene protocols, professional periodontal maintenance, nutritional advices as well as regular professional applications of topical fluorides (varnish) must be implemented in all patients with inborn errors of innate immunity (Box 2). A regular and rigorous screening of the oral cavity is also essential and persistent mucosal lesions should be biopsied considering the potential risk of malignant transformation (e.g., HPV-related oral squamous cell carcinomas in WHIM patients). The treatment of oral ulcers is mainly symptomatic and must include pain management. Indeed, mouth aphthous ulcers may be very painful and cause difficulties in eating, leading to nutritional problems with a potential impact on the patient's general condition. In addition to analgesics, oral film-forming agents containing hyaluronic acid can be used to form a protective barrier over the ulcerated oral mucosa. These protective agents are generally well-tolerated but provide only transient pain relief. In cancer patients with oral mucositis, the use of topical anesthetics such as lidocaine has been recommended for pain management although there are no studies available to assess their benefit and their potential toxicity (272). One should therefore be cautious when advising topical applications of anesthetics in PID patients as they may diminish the swallowing reflex (risk of food aspiration), alter taste sensation with a burning sensation, and be associated with possible cardiovascular effects. In addition, it mostly provides only short-term pain relief (272).

Box 2 **Important Considerations for Oral Management of PID Patients (274, 275).**

- Systematic discussion with the medical team.
- Immediately after the diagnosis of PID: complete oral assessment including clinical and radiographic (at least one panoramic radiography, complemented by appropriate radiographs such as bitewings or cone beam computed tomography) examinations.
- Implementation of intensive preventive measures: individual oral hygiene instructions, nutritional counseling, professional topical fluorides applications.
- Regular follow-up every 3–4 months including periodontal maintenance with professional plaque removal to prevent/limit periodontal inflammation.
- Before initiation of invasive dental procedures: discussion of antibiotic prophylaxis with the medical team (consider the high risk of antibiotic resistance development), complete blood count with a particular attention to neutrophil and platelets counts.
- In case of periodontitis: conventional mechanical periodontal treatment to remove tooth-associated biofilm +/– systemic antibiotics for severe forms, followed by regular supportive periodontal therapy.
- Immediate treatment of oral infections with appropriate antimicrobial agents considering the high risk of invasive infections.

Severe, recurrent and/or persistent oral infections may signal an underlying inborn error of immunity (after exclusion secondary immunodeficiencies). It is crucial to identify the causative pathogen as it gives clue on the signaling pathway that should be explored. For example, germline mutations affecting type I and II IFN signaling pathways should be investigated in patients with severe and relapsing herpetic gingivostomatitis. In a similar way to the management of systemic infections, the treatment of oral infections relies on the administration of antimicrobial agents (e.g., antibiotics, antifungal agents) depending on the type of pathogen that is involved. It should be initiated as soon as an oral infection is suspected considering the high risk of dissemination (Box 2). Since some patients are under continuous antibiotic and/or antifungal curative or prophylactic treatments, one should consider the use of another pharmacological class, after discussion with the medical team, in order to avoid the development of resistances. An antibiogram or even an antifungigram should be performed in order to determine the susceptibility of the causative microorganism and to adapt the treatment subsequently. One study suggested that human polyvalent IV IgGs, administered as a mouthwash, could constitute a novel adjuvant topical treatment of chronic oral candidiasis, in particular in cases of drug resistance, probably through their ability to opsonize *Candid*a (273). Finally, antibiotic prophylaxis must also be discussed with the medical team before invasive dental treatment (i.e., all dental procedures that involve a manipulation of gingival tissue and the periapical region of teeth or induce a perforation of the oral mucosa) and oral surgery to prevent the onset of infections through the entrance way provided by the therapeutic action (Box 2) (274, 275). Pre-operative antibiotic prophylaxis must be systematically administrated in a single dose before invasive oral or dental procedures in patients with associated congenital cardiopathy, such as WHIM patients (214), that are at increased risk to develop infectious endocarditis (276).

Periodontal diseases (i.e., gingivitis and periodontitis) are particularly prevalent in congenital defects of neutrophil number and function. Indeed, neutrophils represent more than 95% of the total number of leukocytes found in the periodontium. Considered as “gatekeepers of oral immunity,” they form the first line of defense against the subgingival biofilm (11). In children and young adults, aggressive periodontitis with early tooth loss almost always indicates the existence of an underlying systemic or immunologic disorder. In some cases, it can even precede the other clinical manifestations. It is therefore crucial to refer the patient for appropriate medical investigation in order to allow a timely diagnosis and the implementation of adequate treatment. The management of the periodontal disease itself relies on careful plaque control with tailored oral hygiene regimens and on conventional mechanical periodontal treatment to remove or at least disrupt tooth-associated biofilm. The latter can possibly be complemented by systemic antibiotics in cases of severe forms of periodontitis, and by antiseptic mouth rinses (e.g., 0.2% chlorhexidine). Regular follow-up (every 3 months) is strongly recommended and must include periodontal maintenance with professional plaque removal (Box 2). The outcome of periodontal treatment in patients with defects of innate immunity seems however unpredictable and independent of the type of treatment provided (65). The highest rate of “stabilization” of the periodontal condition was reported for SCN patients but in only 61% of the cases (vs. less than 43% in other neutrophil-associated PIDs) (65). Unsuccessful outcome suggests that mechanisms other than a defective neutrophil defense against bacteria contribute to the development of periodontal disease (65, 277). Initially, increased host susceptibility to severe periodontitis was thought to be linked to the reduced number or the dysfunction of neutrophils at the gingival sulcus, leading to inefficient control of the periodontal pathogens. However, the implication of immunoregulatory defects responsible for a dysregulated inflammatory response has also been suggested, in particular in PLS syndrome and LAD1 (112). In PLS, despite CTSC deficiency, neutrophil remain capable to destroy pathogens. However, they fail to activate neutrophil proteases, which degrade certain chemokines and cytokines, a process that is crucial for periodontal homeostasis maintenance (112). In LAD1, aggressive periodontitis has been linked to the dysregulation of the IL23/Th17 axis, leading to an increased secretion of IL17 (95, 96). An overproduction of this pro-inflammatory cytokine has also been involved in the initiation and progression of chronic periodontitis, which is the most common form of periodontitis in the general population (98). The study of PID patients with pathogenic variants that alter key effectors of mucosal immunity provides therefore a better understanding of the immune pathways regulating oral mucosal homeostasis (112). The best example is the discovery of the role played by IL17 axis in the maintenance of oral equilibrium. High levels of IL17 lead to enhanced periodontal inflammation as stated above, whereas a decreased generation of Th17 cells and the impairment of IL17/IL23 signaling pathway are associated with a high susceptibility to oral candidiasis (CMC), highlighting the role of IL17 axis in antifungal immunity at barrier sites (250, 252). A better knowledge of the underlying mechanisms also gives clues for the management of these conditions. In moderate forms of LAD1, inhibition of IL23 and IL17 appears to be a promising therapeutic strategy, in particular in cases of severe periodontal involvement (87).

The treatment of the immunological defect itself may allow an improvement of the oral manifestations, especially mucosal lesions. One example is G-CSF therapy that is the “gold standard” treatment for SCN and CyN (13). Indeed, G-CSF administrations have been associated with a decrease of oral ulcers severity and recurrence (78). However, several authors have observed that periodontal disease tends to persist even after normalization of neutrophil counts (67, 70). HSCT provides a definitive correction for most PIDs and remains the only curative treatment for patients with severe forms (278, 279). This procedure therefore also allows an improvement of the lesions affecting the oral mucosa and the gingiva, in particular oral infections, aphthous ulcers and periodontal inflammation. For example, Carlsson et al. reported the case of one patient with SCN1 who did not experience any gingivitis since HSCT (67). However, if gingival inflammation is associated with alveolar bone loss (i.e,. periodontitis) before HSCT, the latter may not be reverted by the procedure. Similarly, preexisting dental developmental anomalies (i.e., alterations in the number, the shape, the size or the structure of the teeth) will not be corrected by HSCT. Despite the improved outcome observed after HSCT, patients still face severe short and long-term transplant-related complications (278, 279). HSCT can therefore also be responsible for several oral complications that include, among others, infections, mucositis, graft-vs.-host disease (GvHD) (280), secondary malignancies (281), and dental sequelae (e.g., agenesis, microdontia, enamel hypoplasia) (272).

Given the low prevalence of inborn errors of innate immunity, orofacial manifestations associated with these conditions have been rarely evaluated in dedicated clinical studies and most of the descriptions arise from case studies. Recently, Halai et al. published the first systematic review on the periodontal status of children with neutrophil associated PIDs. Although 118 studies were included, 98% of them were case reports or case series (65). In addition, these data are often difficult to interpret due to the lack of control groups in most clinical studies. This is particularly true for periodontal diseases that also have a high prevalence in the general population. Several questions regarding orofacial involvement in PIDs remain therefore open, such as the impact of the different oral manifestations on the course of the PID itself. Indeed, the existence of persistent oral infections or periodontal inflammation may contribute to the chronic stimulation of the immune system and also favor the development of secondary autoimmune and inflammatory complications. One should also take into consideration the influence of the treatments. For example, immunosuppressant drugs used in PIDs patients presenting autoimmune manifestations can predispose to the development of oral manifestations, worsen oral infections and affect the progression of periodontal inflammation. The impact of orofacial involvement on the quality of life has also only been poorly investigated, if ever, in PIDs patients and is certainly underestimated.

Further studies are therefore strongly required to better define the orofacial phenotypic spectrums associated with the different inborn errors of innate immunity as well as their impact on the disease course and on the quality of life of the patients. In addition, they will allow to get a better understanding of oral and mucosal immune mechanisms that is a prerequisite for the development of targeted therapeutic strategies.

## Ethics Statement

Informed consents were obtained from the patients for photographs publication and are available at Reference Center for Oral Manifestations (O-Rares) from Strasbourg.

## Author Contributions

SJ designed the study and performed literature search. SJ, VG, AG, and A-SK wrote the paper. SJ prepared the tables. All authors reviewed the manuscript and concur with the submission.

## Conflict of Interest

The authors declare that the research was conducted in the absence of any commercial or financial relationships that could be construed as a potential conflict of interest.

## Abbreviations

AD: autosomal dominant
AML: acute myeloid leukemia
ANC: absolute neutrophil count
AR: autosomal recessive
BCG: Bacille-Calmette-Guérin
CARD9: caspase recruitment domain–containing protein 9
CDG: congenital disorder of glycosylation
CGD: chronic granulomatous disease
CID: combined immunodeficiency
CMC: chronic mucocutaneous candidiasis
CMCD: chronic mucocutaneous candidiasis disease
CMV: cytomegalovirus
CN: congenital neutropenia
CNS: central nervous system
CTSC: cathepsin C
CXCR4: CX chemokine receptor 4
CyN: cyclic neutropenia
DC: dendritic cell
EBV: Epstein Barr Virus
EDA: ectodermal dysplasia
ER: endoplasmic reticulum
EV: epidermodysplasia verruciformis
FPR: formyl peptide receptor
GATA2: guanine-adenine-thymine-adenine 2
GDP: guanosine diphosphate
G-CSF: granulocyte colony-stimulating factor
GI: gastrointestinal
GM-CSF: granulocyte-macrophage colony-stimulating factor
GOF: gain-of-function
G6PC3: glucose-6-phosphatase 3
GvHD: graft-vs.-host disease
HPV: human papillomavirus
HSCT: hematopoietic stem cell transplantation
HSE: herpes simplex virus 1 encephalitis
HSV1: herpes simplex virus 1
IBD: inflammatory bowel disease
IFN: interferon
IFNAR: interferon-α/β receptor
IL: interleukin
IP: incontinentia pigmenti
IRAK-4: IL1 receptor-associated kinase-4
IRF: interferon regulatory factor
IUIS: International Union of Immunological Societies
IVIgs: intravenous immunoglobulins
JAK: Janus kinase
LAD: leukocyte adhesion deficiency
LOF: loss-of-function
MAPK: mitogen-activated protein kinase
MDS: myelodysplastic syndrome
MSMD: Mendelian susceptibility to mycobacterial disease
MyD88: myeloid differentiation factor 88
NADPH: nicotinamide adenine dinucleotide phosphate
NEMO: NF-κB essential modulator
NF-κB: nuclear factor kappa B
NET: neutrophil extracellular trap
NK: natural killer
NOX: NADPH oxidase
NTM: non-tuberculous mycobacteria
PID: primary immunodeficiency
PLS: Papillon-Lefèvre syndrome
ROS: reactive oxygen species
SC: subcutaneous
SCID: severe combined immunodeficiency
SCN: severe congenital neutropenia
SNP: single-nucleotide polymorphism
STAT: signal transducer and activator of transcription
VZV: varicella-zoster virus
WAS: Wiskott-Aldrich syndrome
WASP: Wiskott-Aldrich syndrome protein
XD: X-linked dominant
XR: X-linked recessive
WHIM, warts, hypogammaglobulinemia, infections: myelokathexis. *: refer to lexicon (Table S2).
