# Supplementary material for: Primary Immunodeficiencies With Defects in Innate Immunity: Focus on Orofacial Manifestations
Source: Front Immunol. 2020 Jun 18;11:1065. doi: 10.3389/fimmu.2020.01065 (PMC7314950; doi:10.3389/fimmu.2020.01065)
Supplement: Supplementary file 1 [file Table_1.pdf]

CATEGORY 1. Immunodeficiencies affecting cellular and humoral immunity	T-B+ severe combined immune deficiency
	T-B- SCID
	Combined immunodeficiencies (CID), generally less profound than SCID
CATEGORY 2. Combined immunodeficiencies with associated or syndromic features	Immunodeficiency with congenital thrombocytopenia
	DNA repair defects other than those listed in 1.
	Thymic defects with additional congenital anomalies
	Immuno-osseous dysplasias
	Hyper IgE syndromes
	Defects of vitamin B12 and folate metabolism
	Anhidrotic ectodermodysplasia with immunodeficiency (EDA-ID)
	Calcium channel defects
	Other defects
CATEGORY 3. Predominantly antibody (Ab) deficiencies	Severe reduction in all serum immunoglobulin isotypes with profoundly decreased or absent B cells, agammaglobulinemia
	Severe reduction in at least 2 serum immunoglobulin isotypes with normal or low number of B cells, common variable immunodeficiency (CVID) phenotype
	Severe reduction in serum IgG and IgA with normal/elevated IgM and normal numbers of B cells, hyper IgM
	Isotype, light chain, or functional deficiencies with generally normal numbers of B cells
CATEGORY 4. Diseases of immune dysregulation	Familial hemophagocytic lymphohistiocytosis (FHL)
	FHL syndromes with hypopigmentation
	Regulatory T cell defects
	Autoimmunity with or without lymphoproliferation
	Immune dysregulation with colitis
	Autoimmune lymphoproliferative syndrome (ALPS)
	Susceptibility to EBV and lymphoproliferative conditions
**CATEGORY 5. Congenital defects of phagocyte number or function**	**Congenital neutropenias (CN)**
	**Defects of motility**
	**Defects of respiratory burst**
	**Other non-lymphoid defects**
**CATEGORY 6. Defects in intrinsic and innate immunity**	**Mendelian susceptibility to mycobacterial disease (MSMD)**
	**Epidermodysplasia verruciformis (EV)**
	**Predisposition to severe viral infection**
	**Herpes simplex encephalitis (HSE)**
	**Predisposition to invasive fungal diseases**
	**Predisposition to mucocutaneous candidiasis (CMC)**
	**TLR signaling pathway deficiency with bacterial susceptibility**
	Other inborn errors of immunity related to non-hematopoietic tissues
	Other inborn errors of immunity related to leukocytes
CATEGORY 7. Autoinflammatory diseases	Type 1 interferonopathies
	Defects affecting the inflammasome
	Non-inflammasome-related conditions
CATEGORY 8. Complement deficiencies	
CATEGORY 9. Bone marrow failure	
CATEGORY 10. Phenocopies of inborn errors of immunity	Associated with somatic mutations
	Associated with autoantibodies

Table S1: PID disease categories according to the 2019 classification of the International Union of Immunological Societies (IUIS) (3)

PIDs with defects in innate immunity that are discussed in this review are highlighted in bold.
