# Supplementary material for: Primary Immunodeficiencies With Defects in Innate Immunity: Focus on Orofacial Manifestations
Source: Front Immunol. 2020 Jun 18;11:1065. doi: 10.3389/fimmu.2020.01065 (PMC7314950; doi:10.3389/fimmu.2020.01065)
Supplement: Supplementary file 2 [file Table_2.pdf]

**Agenesis**	Congenital absence of ≥ 1 tooth from the normal series.
**Alveolar bone or alveolar process**	Part of the jaw (mandible or maxilla), which surrounds the roots of the teeth, and forms the tooth sockets.
**Anodontia**	Congenital absence of all the teeth from the normal series.
**Aphthous ulcer**	Ulcer that specifically affects the mucous membranes. It is typically a painful, round or ovoid lesion with a well-defined erythematous margin, like a halo, and a central yellow or gray floor.
**Bowenoid papulosis**	Rare skin disorder linked to HPV infection (pre-cancerous skin condition) and characterized clinically by the presence of scattered papules or small plaques, multiple and pigmented, that involve the stratified squamous epithelium. These lesions have been rarely reported on the oral mucosa.
**Cementum**	Mineralized connective tissue covering the dental root. It allows anchoring of the periodontal ligament's fibers.
**Dental or tooth anomalies/abnormalities**	Alteration in the number, shape, size, or structure, in the chronology of eruption or in the alignment in the dental arch, of a single tooth or multiple teeth.
**Dental plaque or biofilm**	Archetypical biofilm composed of a complex community of micro-organisms embedded in a highly organized intercellular matrix that adheres to the tooth surfaces and to other hard surfaces (e.g. dental restorations, orthodontic appliances, prostheses) in the oral cavity. It is the etiological agent for major dental diseases such as dental caries and periodontal disease*.
**Ectodermal dysplasia (EDA)**	Syndrome characterized by abnormalities of ectodermal structures including anodontia* or oligodontia*, cone shaped teeth, hypotrichosis, hypohidrosis with heat intolerance and due to mutation in ectodysplasin A (EDA) pathway.
**Gingiva or gum**	Part of the oral mucosa covering the alveolar bone.
**Gingival crevice or sulcus**	Natural space found between a tooth and the surrounding gingival tissue called free gingiva. It is lined by sulcular epithelium.
**Gingival crevicular fluid**	Inflammatory exudate derived from the periodontal tissues that contains serum and locally generated constituents (e.g. tissue breakdown products, inflammatory mediators).
**Gingivitis**	Inflammatory lesion which remains confined to the gingiva and does not extend to the periodontal attachment (cementum*, periodontal ligament* and alveolar bone*) and beyond the mucogingival junction. Such inflammation is reversible.
**Gingivostomatitis**	Combination of gingivitis* and stomatitis*.
**Hypodontia**	Congenital absence of ≤ 5 teeth from the normal series.
**Oligodontia**	Congenital absence of ≥ 6 teeth from the normal series.
**Periodontal diseases**	Inflammatory conditions that affect the periodontium* and that include gingivitis* and periodontitis*.
**Periodontal ligament**	Connective tissue attaching the tooth root to the alveolar bone* of the maxilla or the mandible.
**Periodontal pocket**	Pathologically deepened gingival crevice/sulcus*.

**Periodontium**	Specialized tissues that both surround and support the teeth, maintaining them in the maxillary and mandibular bones. It encompasses the cementum*, the periodontal ligament*, the alveolar bone*, and the gingiva*.
**Periodontitis**	Chronic multifactorial inflammatory disease associated with dysbiotic dental plaque biofilm and characterized by progressive destruction of tooth-supporting tissues, including the gingiva, the cementum, the periodontal ligament, and the alveolar bone. Unlike gingivitis, this condition is not reversible.
**Poikiloderma**	Skin condition that consists of areas of hypopigmentation, hyperpigmentation, telangiectasias and atrophy.
**Stomatitis**	Inflammation of oral mucosa.
**Synophris**	Fusion of eyebrows above the bridge of nose.

Table S2: Lexicon.
